# Efficacy and Safety of Probiotics in Irritable Bowel Syndrome: A Systematic Review and Meta-Analysis

**DOI:** 10.3389/fphar.2020.00332

**Published:** 2020-04-03

**Authors:** Bing Li, Li Liang, Huijie Deng, Jinmin Guo, He Shu, Li Zhang

**Affiliations:** Department of Pharmacy, 960th Hospital of the PLA, Jinan, China

**Keywords:** efficacy, safety, irratable bowel syndrome, probiotics, meta-analysis

## Abstract

**Background:**

Irritable bowel syndrome is a functional gastrointestinal disease. Evidence has suggested that probiotics may benefit IBS symptoms. However, clinical trials remain conflicting.

**Aims:**

To implement a systematic review and meta-analysis of clinical trials regarding the efficacy and safety of probiotics for IBS patients.

**Methods:**

We searched for relevant trials in Medline(1966 to Jan 2019), Embase(1974 to Jan 2019), the Cochrane Central Register of Controlled Trials(up to Jan 2019), the ClinicalTrials.gov trials register(up to Jan 2019), and Chinese Biomedical Literature Database(1978 to Jan 2019). Risk ratio (RR) and a 95% confidence interval (CI) were calculated for dichotomous outcomes. Standardized mean difference (SMD) and 95% CI were calculated for continuous outcomes.

**Results:**

A total of 59 studies, including 6,761 patients, were obtained. The RR of the improvement or response with probiotics versus placebo was 1.52 (95% CI 1.32–1.76), with significant heterogeneity (I^2^ = 71%, P < 0.001). The SMD of Probiotics in improving global IBS symptoms vs. Placebo was -1.8(95% CI -0.30 to -0.06), with significant heterogeneity (I^2^ = 65%, P < 0.001). It was impossible to draw a determinate conclusion. However, there were differences in subgroup analyses of probiotics type, dose, treatment duration, and geographic position. Probiotics seem to be safe by the analysis of adverse events(RR = 1.07; 95% CI 0.92–1.24; I^2^ = 0, P = 0.83).

**Conclusion:**

Probiotics are effective and safe for IBS patients. Single probiotics with a higher dose (daily dose of probiotics ≥10^10^) and shorter duration (< 8 weeks) seem to be a better choice, but it still needs more trials to prove it.

## Introduction

Irritable bowel syndrome (IBS) is a common functional gastrointestinal disorder associated with abdominal pain, bloating and altered bowel habits (Drossman et al., 2002). It affects 11% of the world-wide population (Lovell and Ford, 2012). IBS reduces health-related quality of life (HRQOL) (Gralnek et al., 2000; Wang et al., 2012) and leads to a significant economic healthcare burden. Although the exact etiology and pathogenesis underlying IBS are still incompletely understood, studies show that IBS was associated with the gastrointestinal (GI) microbiota, chronic low-grade mucosal inflammation, altered regulation of the gut-brain axis, immune function, visceral hypersensitivity, and psychosocial factors(Parkes et al., 2008; Dupont, 2014; Hayes et al., 2014). Since there is no effective cure for IBS, the treatment focuses on alleviating the particular symptoms. New therapeutic options for IBS include tricyclic antidepressants (Rahimi et al., 2009), spasmolytics (Tack et al., 2016), selective serotonin reuptake inhibitors (Bundeff and Woodis, 2014), lubiprostone (Chang et al., 2016) and linaclotide (Chey et al., 2011), and 5-hydroxytryptamine type-3 antagonists such as ramosetron and alosetron (Andresen et al., 2008). However, current treatments are not very useful or may cause adverse reactions (Trinkley and Nahata, 2014).

Evidence (Durban et al., 2013; Jalanka-Tuovinen et al., 2014) has suggested that intestinal microorganisms play an important role in IBS, as numerous studies have indicated that an irregular composition or metabolic activity of intestinal flora in patients with IBS (Simrén et al., 2013; Spiller et al., 2016; Thijssen et al., 2016; Hod et al., 2017; Shin et al., 2018). Therefore, the regulation of the gut microbiota by probiotics is a promising treatment for IBS (Hyland et al., 2014). Probiotics can improve intestinal ﬂora and limit colonization of pathogenic bacteria (Guarner et al., 2012). Investigators have performed numerous clinical trials to assess the efficacy of probiotics for IBS. However, the conclusions have been controversial. Some trials have suggested that probiotics can improve global IBS symptoms (Lyra et al., 2016). Others have demonstrated no effect (Charbonneau et al., 2013). Several articles have not found an apparent effect of probiotics on global IBS symptoms, but have found improvement of individual IBS symptoms (Sisson et al., 2014). Therefore, we conducted this meta-analysis to examine the efficacy of global IBS symptoms improvement, global symptoms scores, and individual symptom scores, such as abdominal pain and bloating. Additionally, this study evaluated the safety of probiotics.

## Methods

### Search Strategy and Selection Criteria

We included all eligible randomized placebo-controlled, trials (RCTs) of probiotics treatment in adult IBS. We searched Medline(1966 to Jan 2019), Embase(1974 to Jan 2019), the Cochrane Central Register of Controlled Trials(up to Jan 2019), the ClinicalTrials.gov trials register(up to Jan 2019), and Chinese Biomedical Literature Database(CBM) (1978 to Jan 2019) for relevant trials. We used the terms “probiotics” and “irritable bowel syndrome” both as medical subject heading (Mesh) and free text terms. The exact search strategy in Medline was (“probiotics”[MeSH Terms] OR “probiotics”[Title/Abstract]) AND (“irritable bowel syndrome”[MeSH Terms] OR “irritable bowel syndrome”[Title/Abstract]) AND (“randomized controlled trial” [pt] OR “randomized controlled trial” [tiab]).

We used the following eligibility criteria: (1) the studies were randomized controlled trials (RCTs) comparing probiotics with placebo; (2) diagnostic criteria included but were not limited to the Manning criteria, and Rome I, Rome II, or Rome III criteria. We did not exclude trials in which patients were stated to be diagnosed with IBS but no diagnostic criteria were described; (3) the age of participants were ≥ 18 years; (4) minimum treatment duration was 7 days. Studies were excluded if they met: (1) studies with inadequate information; (2) probiotics along with other drugs; (3) control group was not placebo; (4) data were not available after contacting the authors. There were no language limitation. Articles in foreign language were translated as needed.

### Outcome Assessment

The primary outcomes were the efficacy of probiotics on global IBS symptoms improvement or response to therapy. Secondary outcomes involved the effect on global symptoms scores and individual symptom scores, such as abdominal pain and bloating. The safety of probiotics was also evaluated.

### Data Extraction

Two reviewers extracted data from included trials independently. All data was inspected by a third reviewer. Any divergence was solved by consensus. Following data were extracted:author publication year, country, type of IBS(%), diagnostic criteria for IBS, recruitment, sample size, number of male/female, age, probiotic, dosage, duration of therapy, criteria to define symptom improvement or response, and outcomes.

### Assessment of Risk of Bias

Two reviewers performed the assessment of study quality independently. Disagreements were solved by discussion. The risk of bias were evaluated according to the Cochrane handbook (Higgins and Green, 2011). Random sequence generation and allocation concealment(selection bias), blinding of participants and personnel(performance bias), blinding of outcome assessment(detection bias), incomplete outcome data(attrition bias), selective reporting(reporting bias), and other biases were assessed.

### Statistical Analyses

Random effects model was used (Dersimonian and Laird, 1986) to get a conservative estimation for the effect. As dichotomous outcomes, the efficacy on global IBS symptoms improvement or overall symptom response and the safety of probiotics were evaluated by RR(risk ratio) and 95% CIs(confidence intervals). As continuous outcomes, global symptoms scores, and individual symptoms scores were assessed using standardised mean difference (SMD) and corresponding 95% CIs. A negative SMD was defined to indicate beneficial effects of probiotics compared with placebo for outcomes. Subgroup analyses based on probiotic type, dosage, and treatment duration were conducted.

Heterogeneity was tested by I^2^ statistic and the Cochran Q-test. I^2^ ≥ 50 and P < 0.10 were considered as a significant heterogeneity (Higgins et al., 2003). When there was significant heterogeneity, sensitivity analyses were conducted to give possible explanation. Review Manager version 5.3.5 (the Nordic Cochrane Center, Copenhagen, Denmark) was used to obtain forest plots of RRs and SMDs Egger test (Egger et al., 1997) (P < 0.10 defined existence of possible publication bias) and funnel plots was calculated by Stata Statistical Software: Release 13 (StataCorp LP; College Station, TX).

## Result

**Figure 1:**
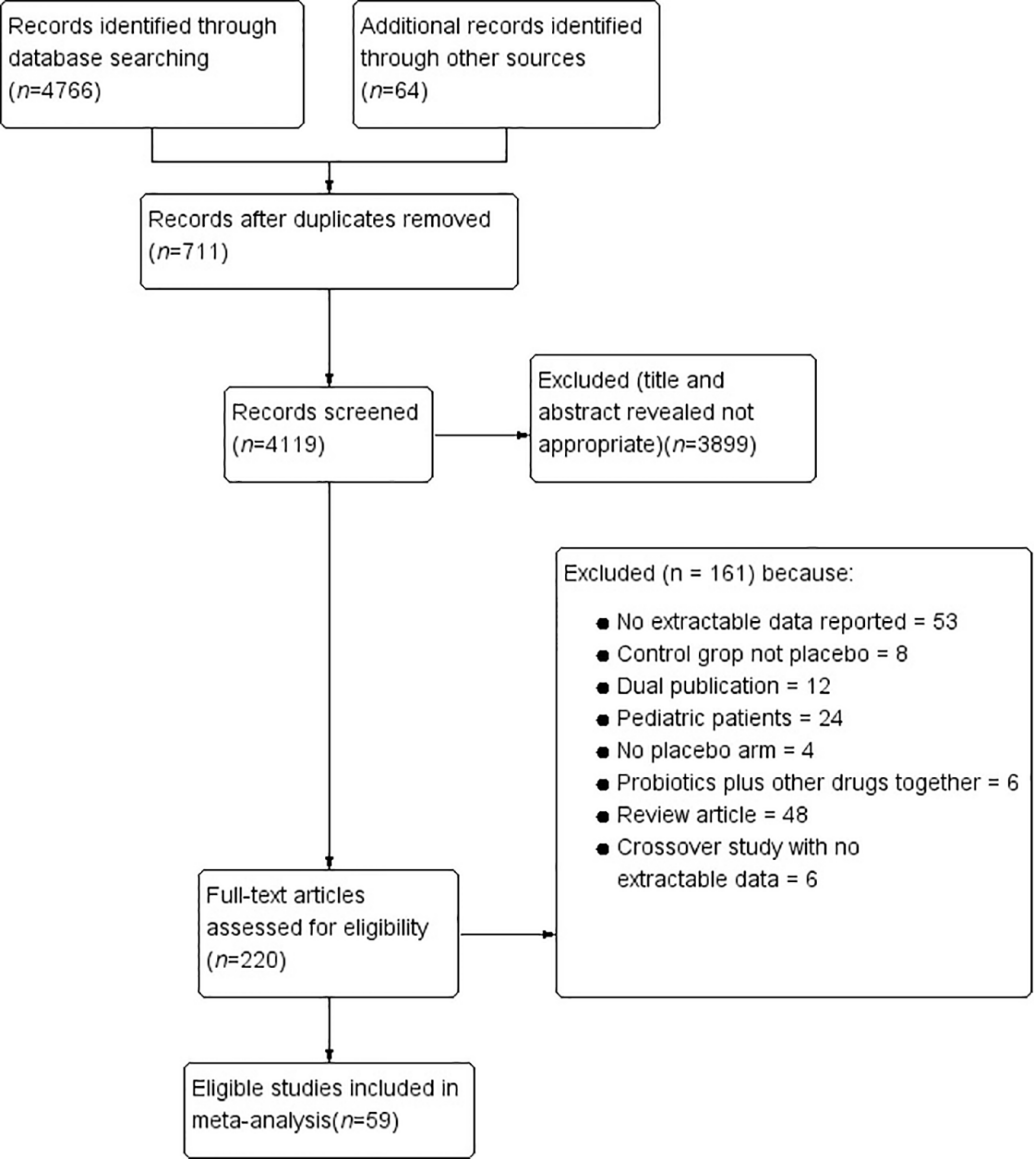
Flow diagram of the study selection process.

**Figure 2:**
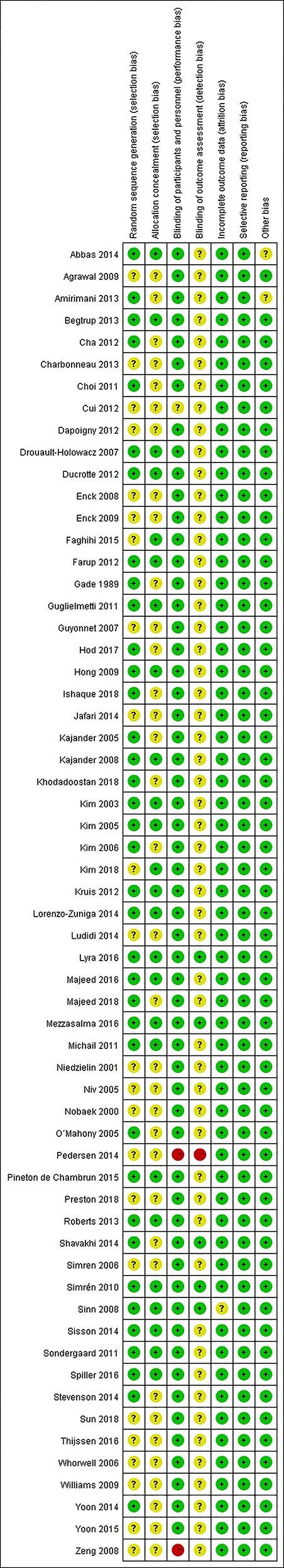
Risk of bias.

**Figure 3:**
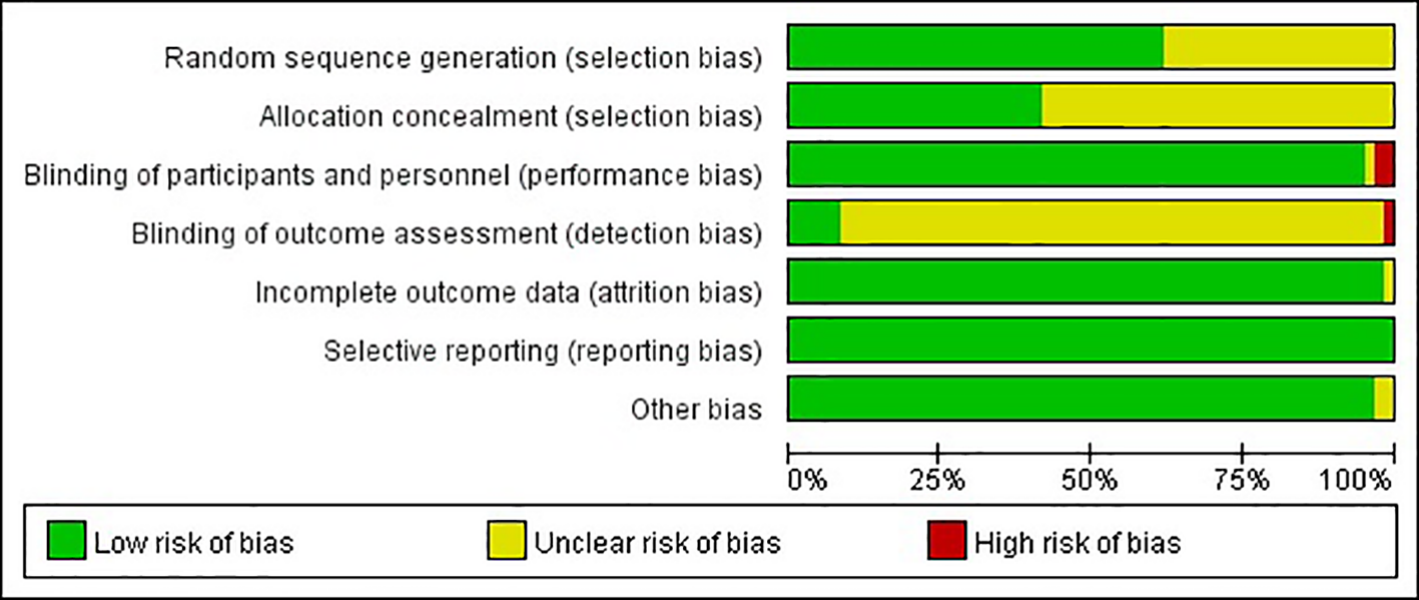
Risk of bias summary.

Based on network searching, a total of 4,830 citations were retrieved. By removing duplicates and screening titles and abstracts, 220 studies remained to be relevant (**Figure 1**). Excluding 161 studies for diverse reasons, 59 studies (Gade and Thorn, 1989; Nobaek et al., 2000; Niedzielin et al., 2001; Kim et al., 2003; Kajander et al., 2005; Kim et al., 2005; Niv et al., 2005; O'Mahony et al., 2005; Kim et al., 2006; Simren and Lindh, 2006; Whorwell et al., 2006; Guyonnet et al., 2007; Drouault-Holowacz et al., 2008; Enck et al., 2008; Kajander et al., 2008; Sinn et al., 2008; Zeng et al., 2008; Agrawal et al., 2009; Enck et al., 2009; Hong et al., 2009; Williams et al., 2009; Simrén et al., 2010; Choi et al., 2011; Guglielmetti et al., 2011; Michail and Kenche, 2011; Sondergaard et al., 2011; Cha et al., 2012; Cui and Hu, 2012; Dapoigny et al., 2012; Ducrotte et al., 2012; Farup et al., 2012; Kruis et al., 2012; Amirimani et al., 2013; Begtrup et al., 2013; Charbonneau et al., 2013; Roberts et al., 2013; Abbas et al., 2014; Jafari et al., 2014; Lorenzo-Zuniga et al., 2014; Ludidi et al., 2014; Pedersen et al., 2014; Shavakhi et al., 2014; Sisson et al., 2014; Stevenson et al., 2014; Yoon et al., 2014; Faghihi et al., 2015; Pineton de Chambrun et al., 2015; Yoon et al., 2015; Lyra et al., 2016; Majeed et al., 2016; Mezzasalma et al., 2016; Spiller et al., 2016; Thijssen et al., 2016; Hod et al., 2017; Ishaque et al., 2018; Khodadoostan et al., 2018; Kim et al., 2018; Preston et al., 2018; Sun et al., 2018), which contained 6,721 participants, were eligible evaluating. The agreement between the two researchers was well established (kappa value = 0.91). The characteristics of the included RCTs are presented in **Table 1**. The risk of bias was shown in **Figure 2** and **Figure 3**. Twenty-three studies did not describe the details of the sequence generation process (Nobaek et al., 2000; Niedzielin et al., 2001; Niv et al., 2005; Simren and Lindh, 2006; Whorwell et al., 2006; Guyonnet et al., 2007; Enck et al., 2008; Zeng et al., 2008; Agrawal et al., 2009; Enck et al., 2009; Williams et al., 2009; Cui and Hu, 2012; Dapoigny et al., 2012; Charbonneau et al., 2013; Jafari et al., 2014; Ludidi et al., 2014; Pedersen et al., 2014; Sisson et al., 2014; Faghihi et al., 2015; Thijssen et al., 2016; Kim et al., 2018; Preston et al., 2018; Sun et al., 2018), and 35 studies did not describe the method of allocation concealment (Gade and Thorn, 1989; Nobaek et al., 2000; Niedzielin et al., 2001; Kajander et al., 2005; Niv et al., 2005; O'Mahony et al., 2005; Kim et al., 2006; Simren and Lindh, 2006; Whorwell et al., 2006; Guyonnet et al., 2007; Enck et al., 2008; Zeng et al., 2008; Agrawal et al., 2009; Enck et al., 2009; Williams et al., 2009; Choi et al., 2011; Cha et al., 2012; Cui and Hu, 2012; Dapoigny et al., 2012; Amirimani et al., 2013; Charbonneau et al., 2013; Jafari et al., 2014; Ludidi et al., 2014; Pedersen et al., 2014; Shavakhi et al., 2014; Stevenson et al., 2014; Yoon et al., 2014; Yoon et al., 2015; Majeed et al., 2016; Thijssen et al., 2016; Hod et al., 2017; Ishaque et al., 2018; Khodadoostan et al., 2018; Preston et al., 2018; Sun et al., 2018), which lead to an unclear risk of selection bias. The risk of blinding the participants and personnel was low, except two studies (Zeng et al., 2008; Pedersen et al., 2014) were at high risk and one (Cui and Hu, 2012) was unclear. The risk of outcome assessment was mostly unclear. However, one study (Pedersen et al., 2014) was an unblinded controlled trial, leading to a high risk of performance and detection bias. Attrition bias, reporting bias, and other biases were low.

**Table 1:** Characteristics of randomized controlled trials of probiotics versus placebo in irritable bowel syndrome.

Study	Year	Country	Type of IBS(%)	diagnostic criteria for IBS	recruitment	Sample size	Sex (Male/Female)		Age[years],mean ± SD	Probiotic	Probiotic dosage(CFU/D)	Duration of therapy	Criteria used to define symptom improvement following therapy or response	Outcome
							**Probiotic**	**Placebo**						
Gade and Thorn (1989)	1989	*Denmark*	all types	Manning	Primary care	54	5/27	7/15	34	Streptococcus faecium	Not stated	4 weeks	IBS symptoms “improved”	Improvement in IBS symptoms Adverse events
Nobaek et al. (2006)	2000	*Sweden*	all types	Rome I	Advertisement	52	9/16	7/20	51	Lactobacillus plantarum	5×10^7^	4 weeks	> 1.5 improvement in VAS scale for abdominal pain, and continuous scale for IBS symptoms	Abdominal pain(VAS)
Niedzielin et al. (2001)	2001	Poland	all types	clinical diagnosis	Primary care	40	5/15	3/17	45	Lactobacillus plantarum	2×10^10^	4 weeks	improvement in IBS symptoms	Improvement in IBS symptoms Adverse events
Kim et al. (2003)	2003	USA	D:100	Rome II	Secondary care	25	2/10	5/8	42.8 ± 16.7	Combination	9×10^11^	8 weeks	Satisfactory relief of IBS symptoms for 50% of weeks, and continuous scale for IBS symptoms	Response(Satisfactory relief of IBS symptoms for 50% of weeks) Overall symptoms score Bloating(100-mm VAS) Abdominal pain(100-mm VAS) Adverse events
Kajander et al. (2005)	2005	Finland	D:48 C:23 A:29	Rome I and II	Advertising	103	13/39	11/40	46	Combination	8–9×10^9^	6 months	Relief of IBS symptoms, and continuous scale for IBS symptoms	Global symptoms score Abdominal pain(a 4-point numerical scale) Adverse events
Kim et al. (2005)	2005	USA	D:42 C:33 A:25	Rome II	Secondary care and advertising	48	3/21	0/24	43	Combination	9×10^11^	4-8 weeks	Satisfactory relief of IBS symptom for 50% of weeks	Response(Satisfactory relief of IBS symptoms for 50% of weeks) Bloating(100-mmVAS) Abdominal pain(100-mmVAS) Adverse events
Niv et al. (2005)	2005	Israel	D:37 C:18.5 M:44.4	Rome II	Secondary care	54	7/20	11/16	45.6	L. reuteri ATCC 55730	4×10^8^ for 1wk, then 2×10^8^	6 months	continuous scale for IBS symptoms	Global symptoms score Adverse events
O'Mahony et al. (2005)	2005	Ireland	D:28 C:26 A:45	Rome II	Secondary care	75	not stated		44.3	L. salivarius UCC4331 or B. infantis 35624	1×10^10^	8 weeks	Continuous scale for IBS symptoms	Global symptoms score Abdominal pain(7-point Likert score) Bloating(7-pointLikert score) Adverse events
Kim et al. (2006)	2006	Korea	D:70 A:30	clinical diagnosis	Secondary care	34	14/3	11/6	39.35 ± 11.9	Combination	3×10^9^(Bacillus subtilis) 2.7×10^10^(Streptococcus faecium)	4 weeks	Continuous scale for IBS symptoms	Bloating(10-pointVAS) Abdominal pain(10-pointVAS) Adverse events
Simren and Lindh (2006)	2006	Sweden	all types	Rome II	Advertising	76	not stated		40	L.plantarum DSM 9843	2×10^10^	6 weeks	Continuous scale for IBS symptoms	Global symptoms score(IBS-SSS)
Whorwell et al. (2006)	2006	UK	D:55.5 C:20.7 A:23.8	Rome II	Primary care	362	0/270	0/92	41.9 ± 10.46	B. infantis 35624	1×10^6^,1×10^8^,1×10^10^	4 weeks	Subjects' Global Assessment (SGA) of IBS symptoms,and continuous scale symptoms for IBS	Response(SGA) Global symptoms score Bloating(a 6-point numerical scale) Abdominal pain(a 6-point numerical scale) Adverse events
Guyonnet et al. (2007)	2007	France	C:100	Rome II	Primary care	267	29/106	39/93	49.3 ± 11.4	Combination	B. animalis DN173010 (1.25×10^10^ c.f.u./125 g) S. thermophilus (1.2×109 c.f.u./125 g) and L. bulgaricus (1.2×109 c.f.u./125 g) b.i.d.	6 weeks	improvement at least 10% vs. baseline	Response(improvement at least 10% vs. baseline) Global symptoms score(a 7-pointLikert score) Bloating(a 7-Likert score) Abdominal pain(a 7-Likert score) Adverse events
Drouault-Holowacz et al. (2007)	2007	France	D:29 C:29 A:41 non-classified:1%	Rome II	Not stated	100	8/40	16/36	45.4 ± 14	Combination	1 × 10^10^	4 weeks	Satisfactory relief of global IBS symptoms	Satisfactory relief of IBS symptoms Abdominal pain(a 4-pointLikert score)
Enck et al. (2008)	2008	Germany	all types	Primary care physicians	Primary care	297	77/72	73/75	49.6 ± 13.6	Enterococcus faecalis DSM16440 and Escherichia coli DSM17252	(3.0-9.0×10^7^c.f.u./1.5 ml)×0.75 ml t.i.d. for 1 week, then 1.5 ml t.i.d. for weeks 2 and 3, then 2.25 ml t.i.d. for weeks 3–8	8 weeks	50% improvement in IBS global symptoms,and continuous scale symptoms for IBS	Response(50% improvement in IBS global symptoms) Global symptoms score(GSS) Adverse events
Kajander et al. (2008)	2008	Finland	D:45 C:30 A:25	Rome II	Primary care	86	2/41	4/39	48 ± 13	Combination	1 × 10^7^	20 weeks	Continuous scale for IBS symptoms	Global symptoms score Flatulence(a 5-point numerical scale) Distension(a 5-point numerical scale) abdominal pain(a 5-point numerical scale) Adverse events
Sinn et al. (2008)	2008	Korea	D:20 C:27.5 M:62.5	Rome III	Secondary care	40	6/14	8/12	44.7 ± 13	L. acidophilus SDC 2012 and 2013	4×10^9^	4 weeks	Any reduction in abdominal pain score	Response(Any reduction in abdominal pain score) Abdominal pain(a 6-point numerical scale) Adverse events
Zeng et al. (2008)	2008	China	D:100	Rome II	Tertiary care	29	10/4	9/6	45.2 ± 10.7	Streptococcus thermophilus, Lactobacillus bulgaricus, Lactobacillus acidophilus and Bifidobacterium Longum	S.thermophilus (4×10^10^ c.f.u.), L. bulgaricus (4×10^9^ c.f.u.), L. acidophilus (4×10^9^ c.f.u.), and B. longum (4×10^9^ c.f.u.)	4 weeks	Continuous scale for IBS symptoms	Global symptoms score Bloating(100-mm VAS) Abdominal pain(100-mm VAS) Adverse events
Agrawal et al. (2009)	2009	UK	C:100	Rome III	Tertiary care	34	0/17	0/17	39.4 ± 10.6	Bifidobacterium lactis DN-173 010 Streptococcus thermophilus and Lactobacillus bulgaricus	B. lactis DN-173 010(2.5×10^10^ c.f.u.), S.thermophilus (2.4×10^9^ c.f.u.), L. bulgaricus (2.4×10^9^ c.f.u.),	4 weeks	Continuous scale for IBS symptoms	Global symptoms score Bloating(a 6-point numerical scale) Flatulence(a 6-point numerical scale) Abdominal pain(a 6-point numerical scale)
Enck et al. (2009)	2009	Germany	All types	Kruis score	Primary care	298	76/72	75/75	49.6 ± 13.6	E. coli DSM17252	(1.5–4.5×10^7^ c.f.u./ml) 0.75 ml drops t.i.d. for 1 week, then 1.5 ml t.i.d. for weeks 2–8	8 weeks	No longer having IBS symptoms	Response(no longer having IBS symptoms) General symptom score Adverse events
Hong et al. (2009)	2009	Korea	D:45.7 C:20 M:8.6 non-classified:25.7	Rome III	tertiary care	70	25/11	22/12	37 ± 14.85	Combination	4×10^10^	8 weeks	Reduction of symptom score by at least 50%	Response(Reduction of symptom score by at least 50%) Adverse events
Williams et al. (2009)	2009	UK	D:11.5C:27 A:61.5	Rome II	Advertising	52	3/25	4/20	39 ± 11.5	Combination	2.5×10^10^	8 weeks	Continuous scale for IBS symptoms	Global symptom score
Simrén et al. (2010)	2010	Sweden	D:35 C:15 M:50	Rome II	Tertiary care	74	11/26	11/26	43 ± 15.43	Combination	2×10^10^	8 weeks	Adequate relief of their IBS symptoms at least 50% of the weeks	Response(Adequate relief of their IBS symptoms) Global symptom score Abdominal pain(100-mm VAS) Bloating(100-mm VAS) Adverse events
Choi et al. (2011)	2011	Korea	D:71.6 M:28.4	Rome II	Tertiary care	90	18/17	19/20	40.4 ± 12.9	Saccharomyces boulardii	4×10^11^	4 weeks	Continuous scale for IBS symptoms	Global symptoms score(7-point Likert scale) Bloating(7-point Likert scale) Abdominal pain(7-point Likert scale) Adverse events
Guglielmetti et al. (2011)	2011	Germany	D:21.3 C:19.7 M:58.2 non-classified:0.8	Rome III	Secondary care and advertising	122	19/41	21/41	38.9 ± 12.75	B. bifidum MIMBb75	1×10^9^	4 weeks	Improvement in average weekly global IBS symptom score of 1 or more for 50% of weeks, and continuous scale for IBS symptoms	Response(Improvement in average weekly global IBS symptom score of 1 or more for 50% of weeks) Global symptoms score(7-point Likert score) Bloating(7-point Likert scale) Abdominal pain(7-point Likert scale) Adverse events
Michail and Kenche (2011)	2011	USA	D:100	Rome III	Tertiary care	24	5/10	3/6	21.8 ± 17	Combination	9×10^11^	8 weeks	Continuous scale for IBS symptoms	Global symptoms score(a clinical rating scale GSRS) Bloating(GSRS) Abdominal pain(GSRS) Adverse events
Sondergaard et al. (2011)	2011	Denmark and Sweden	all types	Rome II	Primary and secondary care	52	7/20	6/19	51.3± 9.5	Combination	2.5×10^10^	8 weeks	Adequate relief of IBS symptoms and continuous scale for IBS symptoms	Adequate relief of IBS symptoms Global symptoms score(IBS SSI Francis et al) Abdominal pain(100-mm VAS)
Cha et al. (2012)	2012	Korea	D:100	Rome III	Tertiary care	50	12/13	14/11	39.1 ± 11.76	Combination	1×10^10^	8 weeks	Adequate relief of their IBS symptoms at least 50% of the weeks and continuous scale for IBS symptoms	Response(Adequate relief of their IBS symptoms at least 50% of the weeks) Global symptoms score(10-point VAS) Abdominal pain(10-point VAS) Bloating(10-point VAS) Adverse events
Cui et al. (2012)	2012	China	D:48.3 C:20 M:11.7 non-classified:10	Rome III	Tertiary care	60	11/26	7/16	44.66 ± 15.23	Combination	1.5×10^7^	4 weeks	reduction of symptom score by at least 30%	Improvement in IBS symptoms
Dapoigny et al. (2012)	2012	France	D:30 C:22 M:34 U:14	Rome III	Tertiary care	50	5/20	10/15	47.05± 10.98	Lactobacillus casei rhamnosus LCR35	6×10^8^	4 weeks	IBS severity score reduced by at least 50%	Response (IBS severity score reduced by at least 50%) Adverse events
Ducrotte et al. (2012)	2012	India	all types	Rome III	Primary care	214	70/38	81/25	37.28± 12.6	L. plantarum LP299V DSM 9843	1×10^11^	4 weeks	Patients rated treatment efficacy as excellent or good	Global assessment of treatment efficacy Adverse events
Farup et al. (2012)	2012	Norway	D:37.5 C:6.25 A:56.25	Rome II	Secondary care	28	Not stated	Not stated	50± 11	L. plantarum MF 1298	1×10^10^	3 weeks	Continuous scale for IBS symptoms	Global symptoms score
Kruis et al. (2012)	2012	Germany	all types	Rome II	Tertiary care	120	12/48	16/44	45.7± 12.4	E. coli Nissle 1917	2.5–25×10^9^ for 4 days then 5–50×10^9^ for 12 weeks	12 weeks	Patients reported contented with treatment	Response (Patients reported contented with treatment) Adverse events
Amirimani et al. (2013)	2013	Iran	all types	Rome III	Secondary care	102	21/32	15/24	41.8± 12.5	Lactobacillus reuteri	1×10^11^	4 weeks	Continuous scale for IBS symptoms	Abdominal pain(questionare) Bloating(questionare) Adverse events
Begtrup et al. (2013)	2013	Denmark	D:40 C:19 M:38 U:2	Rome III	Primary care	131	51/16	46/18	30.52± 9.42	Combination	5.2×10^10^	6 months	Adequate relief of global IBS symptoms for at least 50% of the time, and continuous scale for IBS symptoms	Response (Adequate relief of global IBS symptoms) Global symptoms score Abdominal pain(GSRS-IBS) Bloating(GSRS-IBS) Adverse events
Charbonneau et al. (2013)	2013	Ireland	all types	Rome II	Population based	76	8/31	6/31	45.5± 11	B. infantis 35624	1×10^9^	8 weeks	Continuous scale for IBS symptoms	Global symptom severity(a six-point scale) Abdominal pain((a six-point numerical scale) Bloating(a six-point numerical scale) Adverse events
Roberts et al. (2013)	2013	UK	C and M	ROME III	Primary care	179	13/75	14/77	44.18± 12.36	Bifidobacterium lactis CNCM I-2494 S. thermophilus and L. bulgaricus	2.5×10^10^ 2.4×10^9^ 2.4×10^9^	12 weeks	Subjective global assessment (SGA) of symptom relief	Subjective global assessment (SGA) of symptom relief IBS-SSS Abdominal pain(6 point Likert scale) Bloating(6 point Likert scale)
Abbas et al. (2014)	2014	Pakistan	D:100	Rome III	Tertiary care	72	27/10	26/9	35.4± 11.9	Saccharomyces boulardii	3×10^9^	6 weeks	Continuous scale for IBS symptoms	Abdominal pain(a 4-point scale) Bloating(a 4-point scale) Adverse events
Jafari et al. (2014)	2014	India	all types	Rome III	Secondary care	108	21/33	22/32	36.7± 11.5	Combination	8×10^9^	4 weeks	Satisfactory relief of global IBS symptoms for at least 50% of the time	Relief of IBS symptoms Abdominal pain(100-mm VAS) Bloating(100-mm VAS)
Lorenzo-Zuniga et al. (2014)	2014	Spain	D:100	Rome III	Tertiary care	84	16/39	15/14	46.8± 12.5	Combination	high dose (1-3×10^10^) low dose (3-6×10^9^)	6 weeks	“Considerably relieved” or “completely relieved” of global IBS symptoms for at least 50% of the time	Health-related quality of life(a specifc questionnaire ranging from 1-100) Respond(relief of symptoms) Adverse events
Ludidi et al. (2014)	2014	Netherlands	all types	Rome III	Secondary care and advertising	40	6/15	7/12	40.5± 14.4	Combination	5×10^9^	6 weeks	A 30% or greater improvement in mean symptom composite score(MSS)	Respond(mean symptom composite score MSS)
Pedersen et al. (2014)	2014	Denmark	D:38 C:17.3 A:40.7 non-classified:4	Rome III	Tertiary care	81	14/27	11/29	Not stated	Lactobacillus rhamnosus GG	1.2×10^10^	6 weeks	Continuous scale for IBS symptoms	IBS-SSS
Shavakhi et al. (2014)	2014	Iran	D:32.6 C:45.7 A:21.7	Rome II	Tertiary care	129	20/46	24/39	36.2± 9.2	Combination	2×10^8^	2 weeks	Continuous scale for IBS symptoms	Abdominal pain(a 4-point scale) Distension(a 4-point scale)
Sisson et al. (2014)	2014	UK	D:37.6 C:21.5 M:35.5 U:5.4	Rome III	Primary care and secondary care	186	40/84	17/45	38.3± 10.6	Combination	2×10^8^/kg	12 weeks	Patients reported mild or no symptoms	Respond(IBS-SSS) IBS symptom severity scores (IBS-SSS) Abdominal pain(IBS-SSS) Bloating(IBS-SSS) Adverse events
Stevenson et al. (2014)	2014	South Africa	D:37.6 C:21.5	Rome II	Secondary care	81	2/52	0/27	47.9± 13	Lactobacillus plantarum 299 v	1×10^10^	8 weeks	Continuous scale for IBS symptoms	IBS symptom severity scores (IBS-SSS) Adverse events
Yoon et al. (2014)	2014	Korea	D:53.1 C40.8 M:6.1	Rome III	Tertiary care	49	11/14	6/18	44.5± 14.3	Combination	1×10^10^	4 weeks	Global relief of IBS symptoms	Global relief of IBS symptoms Abdominal pain(a 10-point numerical scale) Bloating(a 10-point numerical scale) Adverse events
Faghihi et al. (2015)	2015	Iran	D:35.3 C39.6 M:25.1	Rome II	Secondary care	139	Not stated	Not stated	38± 13.3	Escherichia coli Nissle 1917	Not stated	6 weeks	Continuous scale for IBS symptoms	Global symptoms score(Birmingham IBS Symptom Questionnaire)
Pineton de Chambrun et al. (2015)	2015	France	D:28.5 C46.9 M:24.6	Rome III	Not stated	179	14/72	11/82	44± 13.3	Saccharomyces cerevisiae CNCM I-3856	4×10^9^	8 weeks	A reduction in the abdominal pain score of 1 arbitrary unit (au) for at least 50% of the time	Improvement in IBS symptoms Abdominal pain(7-point Likert scale) Adverse events
Yoon et al. (2015)	2015	Korea	D:48.1 C:18.5 M:21 U:12.4	Rome III	Tertiary care	80	24/17	19/20	59.3± 12.2	Combination	1×10^10^	4 weeks	Adequate relief of global IBS symptoms	Adequate relief of global IBS symptoms Global symptoms score(10-point VAS) Abdominal pain(10-point VAS) Bloating(10-point VAS)
Lyra et al. (2016)	2016	Finland	D:38.9 C:16.6 M:44 U:0.5	Rome III	Primary care	391	62/198	37/94	47.9± 12.9	L.acidophilus NCFM (ATCC 700396)	low-dose: 1×10^9^ high-dose: 1×10^10^	12 weeks	Continuous scale for IBS symptoms	IBS symptom severity scores (IBS-SSS) Abdominal pain(IBS-SSS) Bloating(IBS-SSS) Adverse events
Majeed et al. (2016)	2016	India	D:100	Rome III	Tertiary care	36	7/11	10/8	35.8± 10.8	Bacillus coagulans MTCC 5856	2×10^9^	90 days	Continuous scale for IBS symptoms	Abdominal pain(Questionnaire) Bloating(Questionnaire) Adverse events
Mezzasalma et al. (2016)	2016	Italy	C:100	Rome III	Not stated	150	Not stated	Not stated	37.4± 12.5	1: L.acidophilus, L. reuteri 2: L.plantarum, L. rhamnosus, B. animalis subsp. Lactis	1: 1×10^10^ 2: 1.5×10^10^	60 days	A decrease of abdominal pain of at least 30% compared to the basal condition for at least 50% of the intervention time	Response(the subject reporting a decrease of symptoms of at least 30% compared to the basal condition for at least 50% of the intervention time)
Spiller et al. (2016)	2016	France	D:20.8 C47.5 M:31.7	Rome III	Primary care and secondary care	379	31/161	31/156	45.3± 14.9	Saccharomyces cerevisiae I-3856	8×10^9^	12 weeks	An improvement of 50% of the weekly average''intestinal pain/discomfort score'' compared with baseline average score for at least 4 out of the last 8 weeks of the study	Response Global symptoms score Abdominal pain(8-point Likert scale) Bloating(8-point Likert scale) Adverse events
Thijssen et al. (2016)	2016	Netherlands	D:30 C:25 A:28.75 U:16.25	Rome II	Secondary care,tertiary care, and advertising	80	13/26	12/29	41.8± 14.1	Lactobacillus casei Shirota	1.3×10^10^	8 weeks	An mean symptom score(MSS) decrease of at least 30%	Response (An mean symptom score(MSS) decrease of at least 30%)
Hod et al. (2017)	2017	Israel	D:100	Rome III	Community and secondary and tertiary care	107	0/54	0/53	Not extractable	Combination	5×10^10^	8 weeks	improvement in symptoms for at least 50% of the tme	Response Adverse events
Ishaque et al. (2018)	2018	Bangladesh	D:100	Rome III	Tertiary care	360	136/45	145/34	31.9 ± 9.9	Combination	8×10^9^	16weeks	Continuous scale for IBS symptoms	IBS symptom severity scores (IBS-SSS) Abdominal pain(IBS-SSS) Adverse events
Khodadoostan et al. (2018)	2018	Iran	D:100	Rome III	Secondary care and tertiary care	67	21/12	22/12	34.1 ± 11.0	Combination	2×10^9^	6 months	Continuous scale for IBS symptoms	Abdominal pain(10-point VAS)
Kim et al. (2018)	2018	Korea	not stated	not stated	Advertising	42	19/11	6/6	32.7 ± 6.6	Lactobacillus gasseri BNR17	low-dose: 1×10^9^ high-dose: 1×10^10^	4 weeks	Continuous scale for IBS symptoms	Abdominal pain(5-point Likert scale) Bloating(5-point Likert scale)
Preston et al. (2018)	2018	USA	D:46.4 C:35.7 M:18.6	Rome III	Tertiary care	113	47/29	21/16	40.4 ± 13.5	Combination	1×10^11^	6 weeks	Continuous scale for IBS symptoms	IBS symptom severity scores (IBS-SSS) Abdominal pain(IBS-SSS) Adverse events
Sun et al. (2018)	2018	China	D:100	Rome III	Tertiary care	200	63/42	53/42	43.9 ± 12.7	Clostridium butyricum	5.67×10^7^	4 weeks	A reduction of ≥50 points of total IBS-SSS score	Response (A reduction of ≥50 points of total IBS-SSS score) IBS symptom severity scores (IBS-SSS) Abdominal pain(IBS-SSS) Bloating(IBS-SSS) Adverse events

### Efficacy of Probiotics on IBS Symptoms Improvement or Response

**Figure 4:**
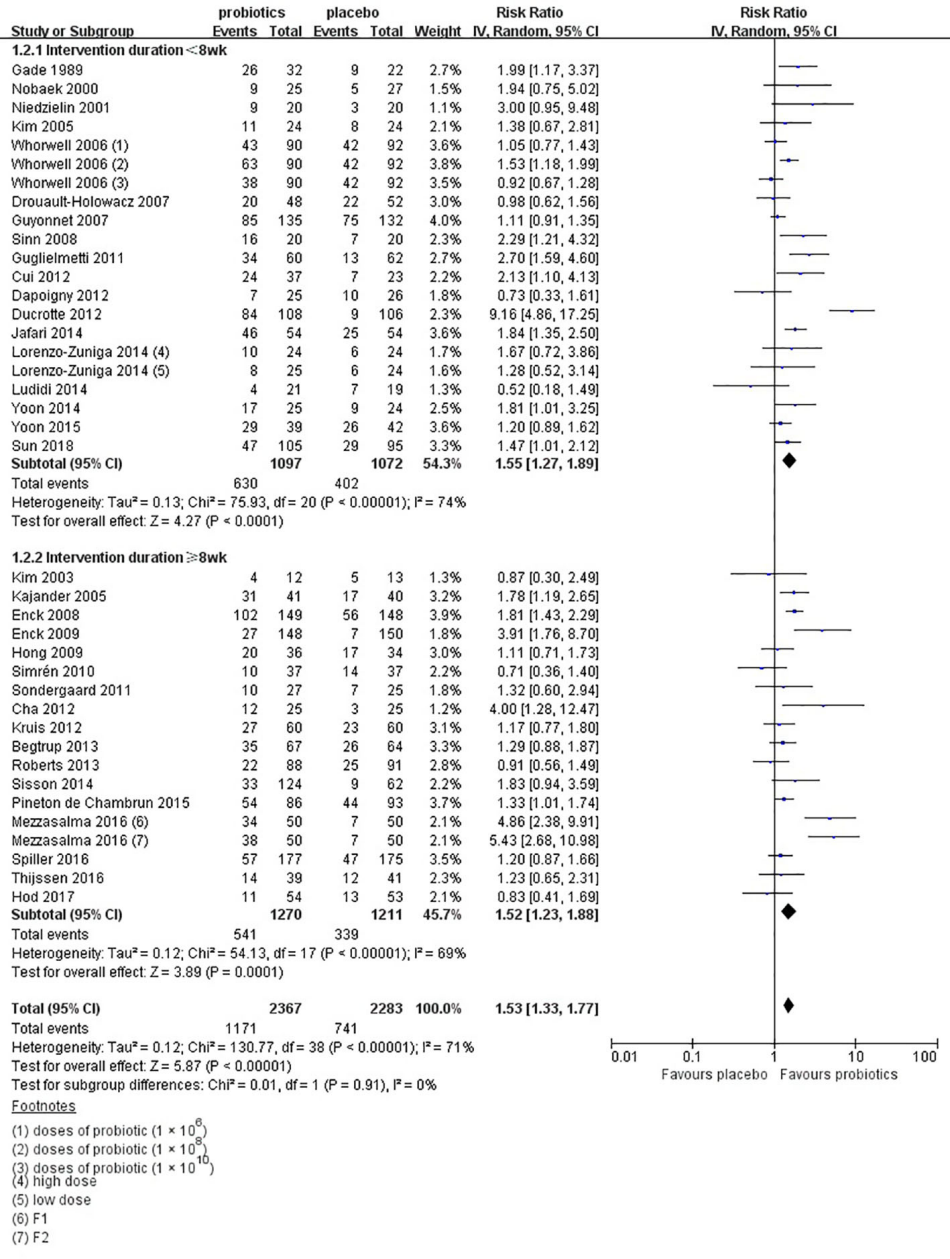
Forest plot of efficacy on IBS symptoms improvement or respond: subgroup of probiotics duration.

Thirty-five RCTs (Gade and Thorn, 1989; Nobaek et al., 2000; Niedzielin et al., 2001; Kim et al., 2003; Kajander et al., 2005; Kim et al., 2005; Whorwell et al., 2006; Guyonnet et al., 2007; Drouault-Holowacz et al., 2008; Enck et al., 2008; Sinn et al., 2008; Enck et al., 2009; Hong et al., 2009; Simrén et al., 2010; Guglielmetti et al., 2011; Sondergaard et al., 2011; Cha et al., 2012; Cui and Hu, 2012; Dapoigny et al., 2012; Ducrotte et al., 2012; Kruis et al., 2012; Begtrup et al., 2013; Roberts et al., 2013; Jafari et al., 2014; Lorenzo-Zuniga et al., 2014; Ludidi et al., 2014; Sisson et al., 2014; Yoon et al., 2014; Pineton de Chambrun et al., 2015; Yoon et al., 2015; Spiller et al., 2016; Mezzasalma et al., 2016; Thijssen et al., 2016; Hod et al., 2017; Sun et al., 2018) with 4,392 patients reported overall IBS symptoms improvement or response as a dichotomous outcome. There were two (Lorenzo-Zuniga et al., 2014; Mezzasalma et al., 2016) of these RCTs examining two different dose groups and one (Whorwell et al., 2006) examining three different dose groups. One (Gade and Thorn, 1989) RCT did not mention the dose of probiotics, so it was not included in the subgroup analysis of probiotics dose. Overall, 1,171(49.5%) of 2,367 patients in the group of probiotics declared symptoms improvement or response after therapy, compared with 644 (31.8%) of 2,025 in the placebo group. The RR of IBS symptoms improvement or response was 1.52(95% CI 1.32–1.76), with high heterogeneity (I^2^ = 71%, P < 0.001; **Figure 4**). The funnel plot suggested the existence of asymmetry (Egger test, P = 0.094; **Figure S1**), indicating possible publication bias. While 19 RCTs (Kim et al., 2003; Kajander et al., 2005; Kim et al., 2005; Drouault-Holowacz et al., 2008; Sinn et al., 2008; Hong et al., 2009; Simrén et al., 2010; Guglielmetti et al., 2011; Sondergaard et al., 2011; Ducrotte et al., 2012; Kruis et al., 2012; Begtrup et al., 2013; Roberts et al., 2013; Sisson et al., 2014; Lorenzo-Zuniga et al., 2014; Pineton de Chambrun et al., 2015; Mezzasalma et al., 2016; Spiller et al., 2016; Hod et al., 2017) with low bias risk were assessed, the effect was still significant (RR = 1.59; 95% CI 1.25–2.04).

**Figure 5:**
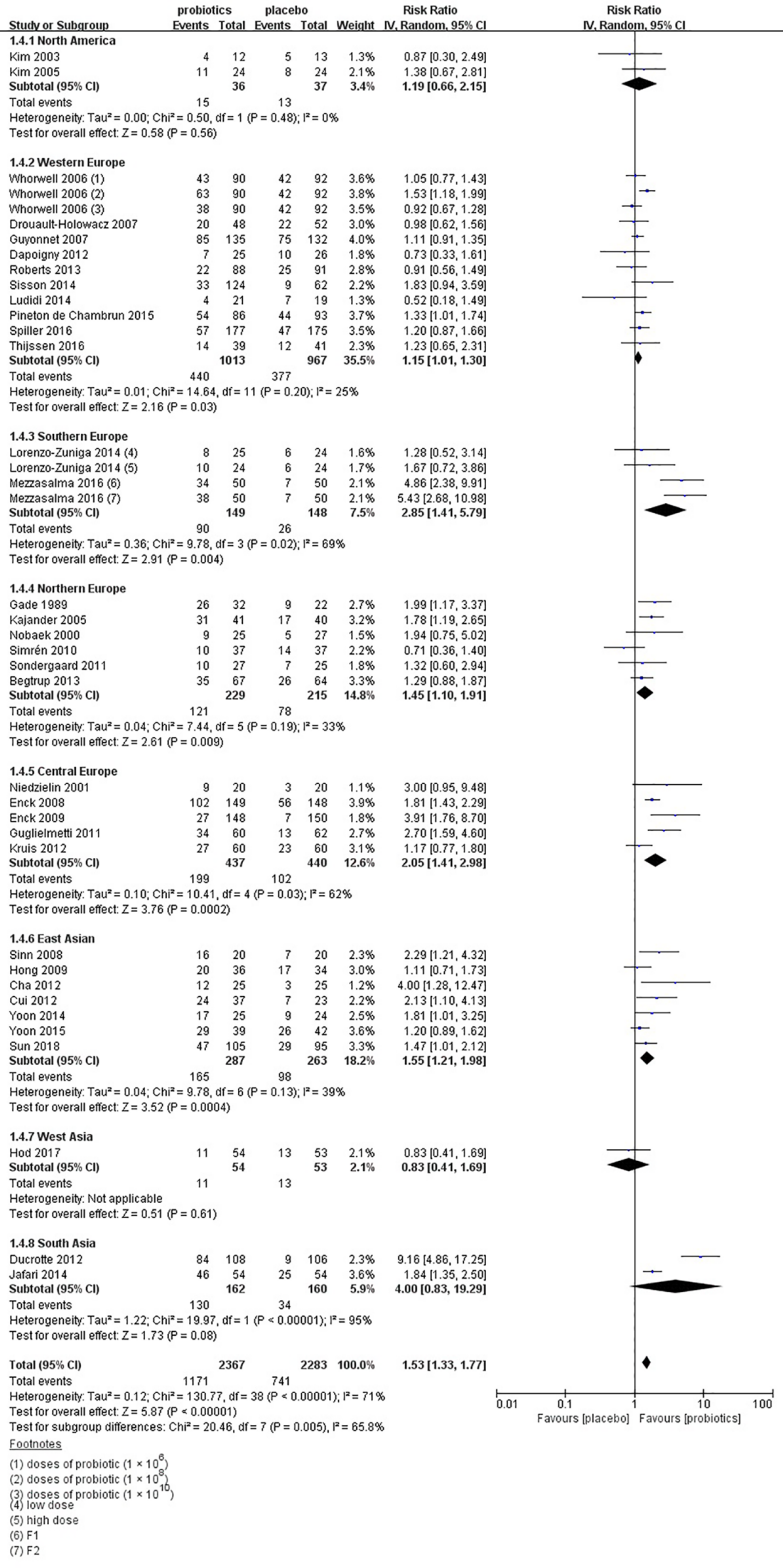
Forest plot of efficacy of probiotics on IBS symptoms improvement or respond: subgroup of geographic position.

In the subgroup of duration, 18 studies (Gade and Thorn, 1989; Nobaek et al., 2000; Niedzielin et al., 2001; Kim et al., 2005; Whorwell et al., 2006; Guyonnet et al., 2007; Drouault-Holowacz et al., 2008; Sinn et al., 2008; Guglielmetti et al., 2011; Cui and Hu, 2012; Dapoigny et al., 2012; Ducrotte et al., 2012; Jafari et al., 2014; Lorenzo-Zuniga et al., 2014; Ludidi et al., 2014; Yoon et al., 2014; Yoon et al., 2015; Sun et al., 2018) evaluated a shorter duration (< 8 weeks) and 17 studies (Kim et al., 2003; Kajander et al., 2005; Enck et al., 2008; Enck et al., 2009; Hong et al., 2009; Simrén et al., 2010; Sondergaard et al., 2011; Cha et al., 2012; Kruis et al., 2012; Begtrup et al., 2013; Roberts et al., 2013; Sisson et al., 2014; Pineton de Chambrun et al., 2015; Mezzasalma et al., 2016; Spiller et al., 2016; Thijssen et al., 2016; Hod et al., 2017) used a longer duration (≥ 8 weeks). The RR of group with less than 8 weeks was 1.55 (95% CI 1.27–1.89; **Figure 4**), and the RR of group with more than 8 weeks was 1.52 (95% CI 1.23–1.88), with significant heterogeneity (I^2^ = 74%, P < 0.01; I^2^ = 69%, P < 0.01, respectively). In the subgroup of probiotics dose, high doses (daily dose of probiotics ≥ 10^10^) were assessed in 21 trials (Niedzielin et al., 2001; Kim et al., 2003; Kim et al., 2005; Whorwell et al., 2006; Guyonnet et al., 2007; Drouault-Holowacz et al., 2008; Hong et al., 2009; Simrén et al., 2010; Sondergaard et al., 2011; Cha et al., 2012; Ducrotte et al., 2012; Kruis et al., 2012; Begtrup et al., 2013; Roberts et al., 2013; Sisson et al., 2014; Lorenzo-Zuniga et al., 2014; Yoon et al., 2014; Yoon et al., 2015; Mezzasalma et al., 2016; Thijssen et al., 2016; Hod et al., 2017). A significant effect on symptoms (RR = 1.51; 95% CI 1.20–1.91; **Figure S2**) and statistically significant heterogeneity (I^2^ = 77%, P < 0.01) were suggested. Low doses (daily dose of probiotics < 10^10^) were evaluated in 15 trials (Nobaek et al., 2000; Kajander et al., 2005; Whorwell et al., 2006; Enck et al., 2008; Sinn et al., 2008; Enck et al., 2009; Guglielmetti et al., 2011; Cui and Hu, 2012; Dapoigny et al., 2012; Jafari et al., 2014; Lorenzo-Zuniga et al., 2014; Ludidi et al., 2014; Pineton de Chambrun et al., 2015; Spiller et al., 2016; Sun et al., 2018). A significant effect on symptoms (RR = 1.56; 95% CI 1.33–1.83) and significant heterogeneity were also detected (I^2^ = 54%, P < 0.01). In the subgroup of probiotics type, there were 15 studies using single probiotics (Gade and Thorn, 1989; Nobaek et al., 2000; Niedzielin et al., 2001; Whorwell et al., 2006; Sinn et al., 2008; Enck et al., 2009; Guglielmetti et al., 2011; Dapoigny et al., 2012; Ducrotte et al., 2012; Kruis et al., 2012; Pineton de Chambrun et al., 2015; Mezzasalma et al., 2016; Spiller et al., 2016; Thijssen et al., 2016; Sun et al., 2018) and 21 studies using combination probiotics (Kim et al., 2003; Kajander et al., 2005; Kim et al., 2005; Guyonnet et al., 2007; Drouault-Holowacz et al., 2008; Enck et al., 2008; Hong et al., 2009; Simrén et al., 2010; Sondergaard et al., 2011; Cha et al., 2012; Cui and Hu, 2012; Begtrup et al., 2013; Roberts et al., 2013; Jafari et al., 2014; Lorenzo-Zuniga et al., 2014; Ludidi et al., 2014; Sisson et al., 2014; Yoon et al., 2014; Yoon et al., 2015; Mezzasalma et al., 2016; Hod et al., 2017). The RR of single and combination group was 1.76 (95% CI 1.37–2.25; **Figure S3**) and 1.39 (95% CI 1.18–1.65), respectively. The I^2^ of the single probiotics subgroup was 69% (P < 0.01), and combination probiotics subgroup was 60% (P < 0.01), suggesting statistically significant heterogeneity. In the subgroup of geographic position, we assigned 2 trials (Kim et al., 2003; Kim et al., 2005) in USA to the North America group; five comparisons of three separate papers (Whorwell et al., 2006; Roberts et al., 2013; Sisson et al., 2014) in UK, five trials (Guyonnet et al., 2007; Drouault-Holowacz et al., 2008; Dapoigny et al., 2012; Pineton de Chambrun et al., 2015; Spiller et al., 2016) in France, and two trials (Ludidi et al., 2014; Thijssen et al., 2016) in Netherlands to the Western Europe group; two comparisons of one papers (Lorenzo-Zuniga et al., 2014) in Spain and two comparisons of one papers (Mezzasalma et al., 2016) in Italy to the South Europe group; two trials (Gade and Thorn, 1989; Begtrup et al., 2013) in Denmark, two trials (Nobaek et al., 2000; Simrén et al., 2010) in Sweden, one trials (Sondergaard et al., 2011) in Denmark and Sweden, and one trials (Kajander et al., 2005) in Finland to the Northern Europe group; one trials (Niedzielin et al., 2001) in Poland and four trials (Enck et al., 2008; Enck et al., 2009; Guglielmetti et al., 2011; Kruis et al., 2012) in Germany to the Central Europe group; five trials (Sinn et al., 2008; Hong et al., 2009; Cha et al., 2012; Yoon et al., 2014; Yoon et al., 2015) in Korea and two trials (Cui and Hu, 2012; Sun et al., 2018) in China to the East Asian group; one trials (Hod et al., 2017) in Israel to the West Asian group; and two trials (Ducrotte et al., 2012; Jafari et al., 2014) in India to the South Asian group. There was a statistically significant benefit in favor of probiotics in North America group (RR = 1.19; 95% CI 0.66–2.15; **Figure 5**), with no significant heterogeneity noted between the studies(I^2^ = 0%, P = 0.48), West Europe group(RR = 1.15; 95% CI 1.01–1.30; I^2^ = 25%, P = 0.20), Northern Europe group(RR = 1.45; 95% CI 1.10–1.91; I^2^ = 33%, P = 0.19) and East Asian group(RR = 1.55; 95% CI 1.21–1.98; I^2^ = 39%, P = 0.13).

### Efficacy of Probiotics on Global IBS Symptoms Scores

**Figure 6:**
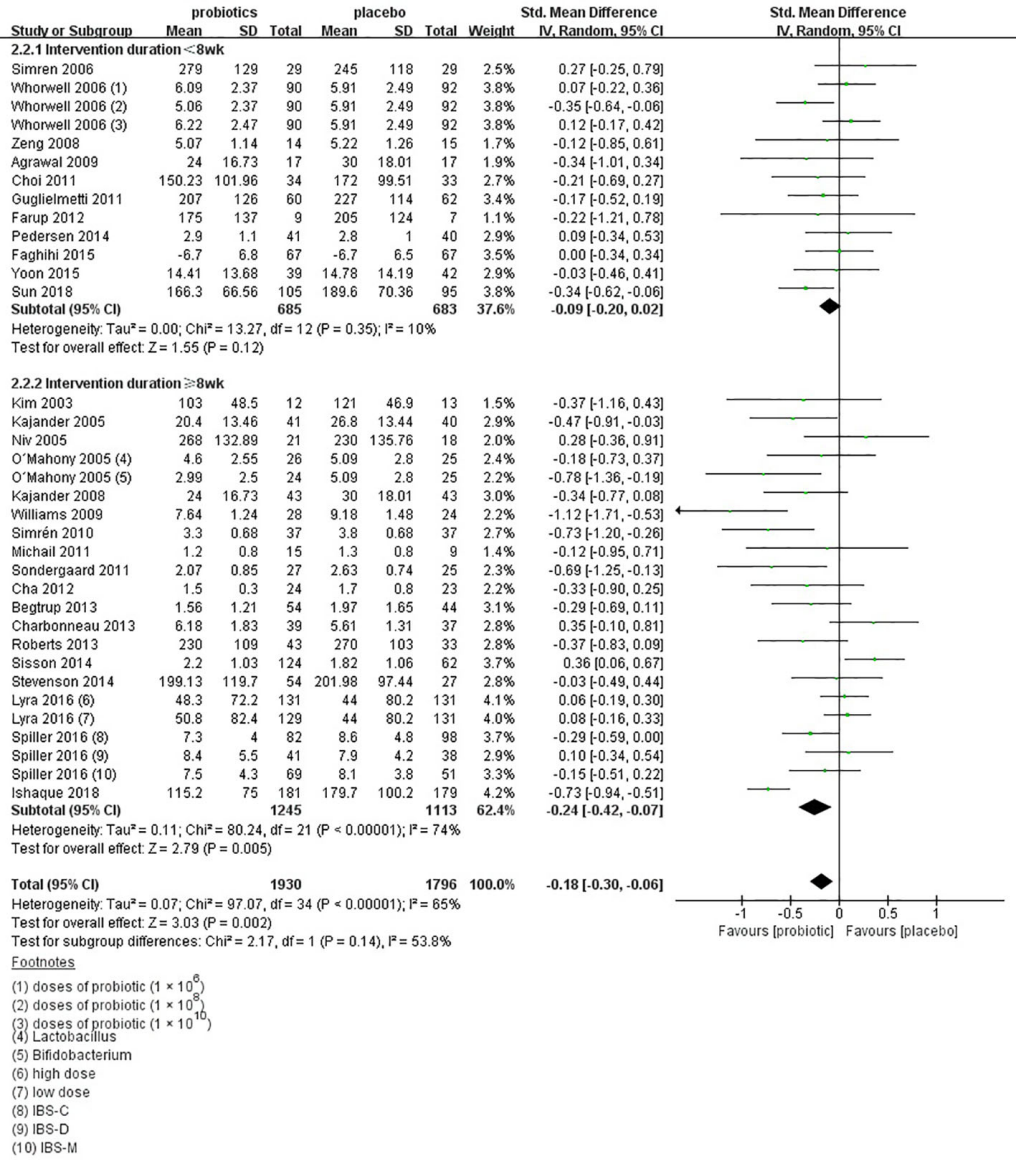
Forest plot of efficacy on global IBS symptoms scores: subgroup of probiotics duration.

There were 29 separate trials (Kim et al., 2003; Kajander et al., 2005; Niv et al., 2005; O'Mahony et al., 2005; Simren and Lindh, 2006; Whorwell et al., 2006; Kajander et al., 2008; Zeng et al., 2008; Agrawal et al., 2009; Williams et al., 2009; Simrén et al., 2010; Choi et al., 2011; Guglielmetti et al., 2011; Michail and Kenche, 2011; Sondergaard et al., 2011; Cha et al., 2012; Farup et al., 2012; Charbonneau et al., 2013; Begtrup et al., 2013; Roberts et al., 2013; Sisson et al., 2014; Pedersen et al., 2014; Stevenson et al., 2014; Faghihi et al., 2015; Yoon et al., 2015; Lyra et al., 2016; Spiller et al., 2016; Ishaque et al., 2018; Sun et al., 2018) including 35 comparisons with 3,726 patients reporting the efficacy of probiotics on global IBS symptoms scores. One (Spiller et al., 2016) of these RCTs examining two different dose groups and one (Whorwell et al., 2006) examining three different dose groups. There was a trial (Faghihi et al., 2015) did not mention the dose of probiotics, so it was not included in the subgroup analysis of probiotics dose. Two types of probiotics were used in one trial (O'Mahony et al., 2005), and three subtypes of IBS, including IBS with diarrhea (IBS-D), IBS with constipation (IBS-C), and IBS with mixed patterns of constipation and diarrhea (IBS-M), were detected separately in one RCT (Spiller et al., 2016). Probiotics had a statistically significant effect on improving the global IBS symptoms vs. placebo (SMD = -1.8; 95% CI -0.30 to -0.06; **Figure 6**). Heterogeneity was significant (I^2^ = 65%, P < 0.001). There was no significant asymmetry in funnel plot (Egger test, P = 0.689; **Figure S4**), indicating no proof of publication bias.

**Figure 7:**
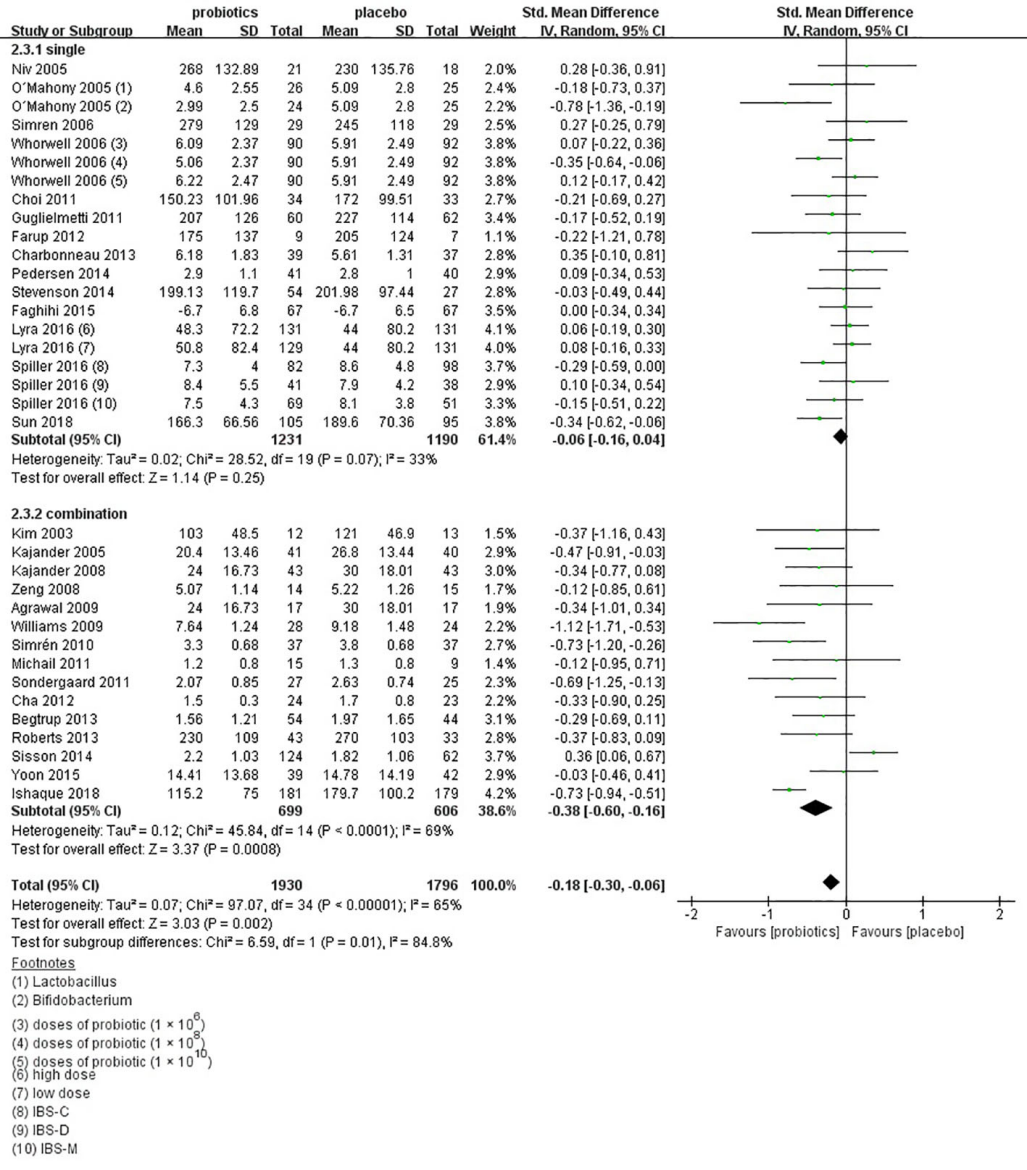
Forest plot of efficacy on global IBS symptoms scores: subgroup of probiotics type.

**Figure 8:**
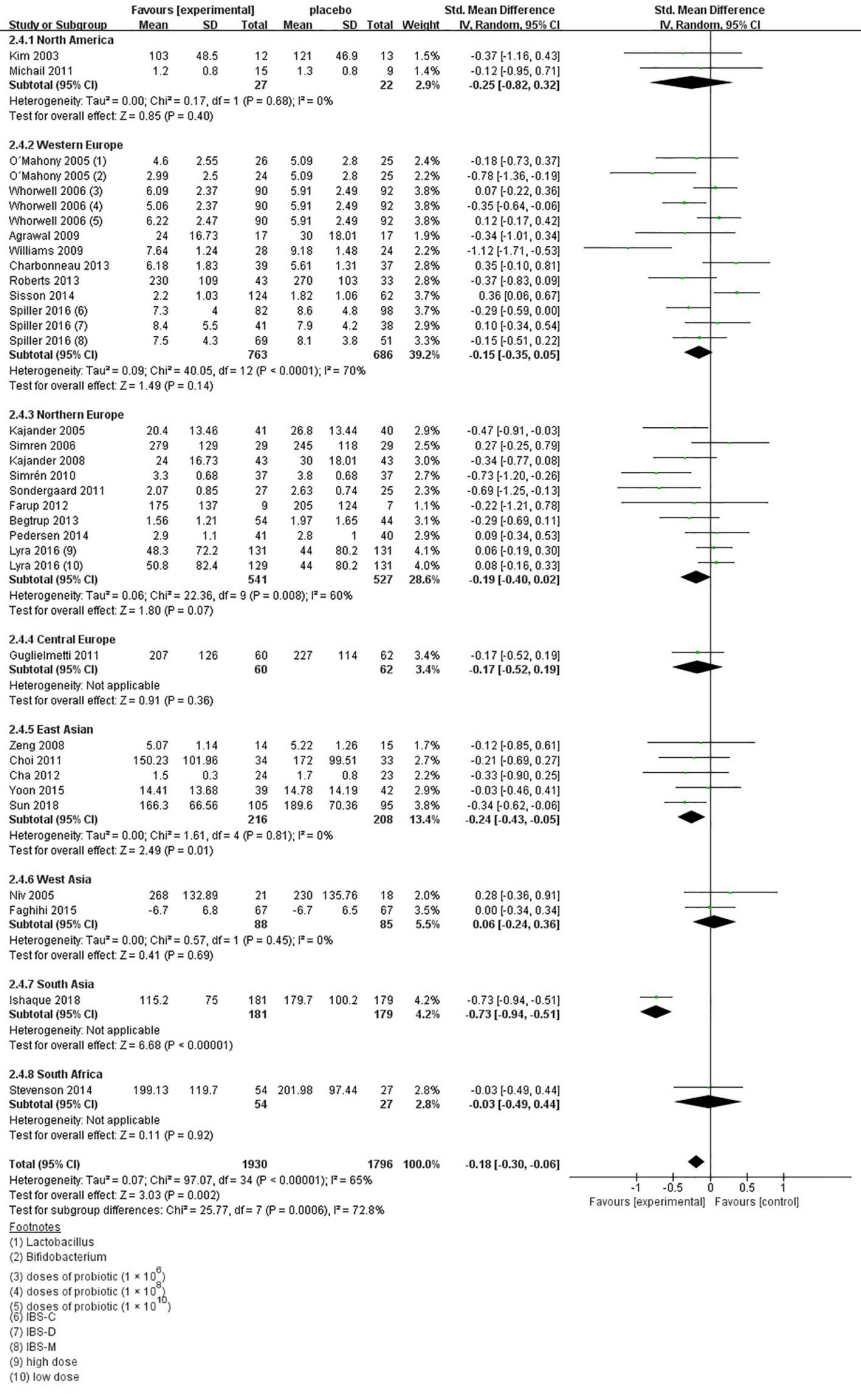
Forest plot of efficacy of probiotics on global IBS symptoms scores: subgroup of geographic position.

In the subgroup of duration, 11 comparisons (Simren and Lindh, 2006; Whorwell et al., 2006; Zeng et al., 2008; Agrawal et al., 2009; Choi et al., 2011; Guglielmetti et al., 2011; Farup et al., 2012; Pedersen et al., 2014; Faghihi et al., 2015; Yoon et al., 2015; Sun et al., 2018) evaluated a shorter treatment duration (< 8 weeks). There was a beneficial effect on global IBS symptoms scores with probiotics (SMD -0.09; 95% CI -0.20 to 0.02) and low heterogeneity was found (I^2^ = 10%, P = 0.12). In the subgroup of probiotics dose, no significant differences were found, as shown in **Figure S5**. In the subgroup of probiotics type, 14 comparisons (Niv et al., 2005; O'Mahony et al., 2005; Simren and Lindh, 2006; Whorwell et al., 2006; Choi et al., 2011; Guglielmetti et al., 2011; Farup et al., 2012; Charbonneau et al., 2013; Pedersen et al., 2014; Stevenson et al., 2014; Faghihi et al., 2015; Lyra et al., 2016; Spiller et al., 2016; Sun et al., 2018) using single probiotics were found a beneficial efficacy on global IBS symptoms scores (SMD -0.06; 95% CI -0.16 to 0.14; **Figure 7**), with low heterogeneity (I^2^ = 33%, P = 0.12). In the subgroup of geographic position, we assigned 2 trials (Kim et al., 2003; Michail and Kenche, 2011) in USA to the North America group; seven comparisons of five separate papers (Whorwell et al., 2006; Agrawal et al., 2009; Williams et al., 2009; Roberts et al., 2013; Sisson et al., 2014) in UK, three comparisons of 1 papers (Spiller et al., 2016) in France, and three comparisons of two separate papers (O'Mahony et al., 2005; Charbonneau et al., 2013) in Ireland to the Western Europe group; two trials (Begtrup et al., 2013; Pedersen et al., 2014) in Denmark, two trials (Simren and Lindh, 2006; Simrén et al., 2010) in Sweden, one trials (Sondergaard et al., 2011) in Denmark and Sweden, four comparisons of three papers (Kajander et al., 2005; Kajander et al., 2008; Lyra et al., 2016) in Finland, and one trials (Farup et al., 2012) in Norway to the Northern Europe group; one trials (Guglielmetti et al., 2011) in Germany to the Central Europe group; three trials (Choi et al., 2011; Cha et al., 2012; Yoon et al., 2015) in Korea and two trials (Zeng et al., 2008; Sun et al., 2018) in China to the East Asian group; one trials (Niv et al., 2005) in Israel and one trials (Faghihi et al., 2015) in Iran to the West Asian group; and one trials (Ishaque et al., 2018) in Bangladesh to the South Asian group; and one trials (Stevenson et al., 2014) in South Africa to the South Africa group. There was a statistically significant benefit in favor of probiotics in North America group (SMD -0.25; 95% CI -0.82 to 0.32; **Figure 8**), with no significant heterogeneity noted between the studies (I^2^ = 0%, P = 0.68), East Asian group (SMD -0.24; 95% CI -0.43 to -0.05; I^2^ = 0%, P = 0.81), and South Asian group (SMD 0.06; 95% CI -0.24 to 0.36; I^2^ = 0%, P = 0.45).

### Efficacy of Probiotics on Individual Symptom Scores

There were 38 trials (Nobaek et al., 2000; Kim et al., 2003; Kajander et al., 2005; Kim et al., 2005; O'Mahony et al., 2005; Kim et al., 2006; Whorwell et al., 2006; Guyonnet et al., 2007; Drouault-Holowacz et al., 2008; Kajander et al., 2008; Sinn et al., 2008; Zeng et al., 2008; Agrawal et al., 2009; Williams et al., 2009; Simrén et al., 2010; Choi et al., 2011; Guglielmetti et al., 2011; Michail and Kenche, 2011; Sondergaard et al., 2011; Cha et al., 2012; Amirimani et al., 2013; Begtrup et al., 2013; Charbonneau et al., 2013; Roberts et al., 2013; Abbas et al., 2014; Jafari et al., 2014; Shavakhi et al., 2014; Sisson et al., 2014; Yoon et al., 2014; Pineton de Chambrun et al., 2015; Yoon et al., 2015; Spiller et al., 2016; Lyra et al., 2016; Majeed et al., 2016; Ishaque et al., 2018; Khodadoostan et al., 2018; Kim et al., 2018; Sun et al., 2018) including 44 comparisons with 4,579 patients reporting efficacy of probiotics on abdominal pain. Probiotics had effect on improving abdominal pain (SMD -0.22; 95% CI -0.33 to -0.11; **Figure S6**), but significant heterogeneity existed (I^2^ = 70%, P < 0.001). However, in subgroup analysis of probiotics dose, 24 comparisons (Kim et al., 2003; Kim et al., 2005; O'Mahony et al., 2005; Kim et al., 2006; Whorwell et al., 2006; Guyonnet et al., 2007; Drouault-Holowacz et al., 2008; Zeng et al., 2008; Agrawal et al., 2009; Williams et al., 2009; Simrén et al., 2010; Choi et al., 2011; Michail and Kenche, 2011; Sondergaard et al., 2011; Cha et al., 2012; Amirimani et al., 2013; Begtrup et al., 2013; Roberts et al., 2013; Sisson et al., 2014; Yoon et al., 2014; Yoon et al., 2015; Lyra et al., 2016; Kim et al., 2018) using high dose were found a significant benefit over placebo (SMD = -0.14; 95% CI -0.26 to -0.01; **Figure S7**), with low heterogeneity (I^2^ = 39%, P = 0.03). There was no significant asymmetry in funnel plot (Egger test, P = 0.235; **Figure S8**), indicating no proof of publication bias.

Twenty-nine trials (Nobaek et al., 2000; Kim et al., 2003; Kim et al., 2005; O'Mahony et al., 2005; Kim et al., 2006; Whorwell et al., 2006; Guyonnet et al., 2007; Zeng et al., 2008; Agrawal et al., 2009; Williams et al., 2009; Simrén et al., 2010; Choi et al., 2011; Guglielmetti et al., 2011; Michail and Kenche, 2011; Cha et al., 2012; Amirimani et al., 2013; Begtrup et al., 2013; Charbonneau et al., 2013; Roberts et al., 2013; Sisson et al., 2014; Abbas et al., 2014; Jafari et al., 2014; Yoon et al., 2014; Yoon et al., 2015; Majeed et al., 2016; Lyra et al., 2016; Spiller et al., 2016; Kim et al., 2018; Sun et al., 2018) reported continuous data for the effect of probiotics on bloating scores in 3,496 patients. Probiotics had effect on improving bloating (SMD -0.13; 95% CI -0.24 to -0.03; **Figure S9**) and heterogeneity was found (I^2^ = 54%, P < 0.01). In the subgroup of probiotics duration, 19 comparisons (Nobaek et al., 2000; Kim et al., 2006; Whorwell et al., 2006; Guyonnet et al., 2007; Zeng et al., 2008; Agrawal et al., 2009; Choi et al., 2011; Guglielmetti et al., 2011; Amirimani et al., 2013; Abbas et al., 2014; Jafari et al., 2014; Yoon et al., 2014; Yoon et al., 2015; Kim et al., 2018; Sun et al., 2018) using a short treatment duration (<8 weeks) were found a significant benefit over placebo (SMD -0.13; 95% CI -0.27 to -0.01). Low heterogeneity was detected (I^2^ = 47%, P = 0.01).There was a beneficial effect on bloating in 22 comparisons (Kim et al., 2003; Kim et al., 2005; O'Mahony et al., 2005; Kim et al., 2006; Whorwell et al., 2006; Guyonnet et al., 2007; Zeng et al., 2008; Agrawal et al., 2009; Williams et al., 2009; Simrén et al., 2010; Choi et al., 2011; Michail and Kenche, 2011; Cha et al., 2012; Amirimani et al., 2013; Begtrup et al., 2013; Roberts et al., 2013; Sisson et al., 2014; Yoon et al., 2014; Yoon et al., 2015; Lyra et al., 2016; Kim et al., 2018) using high dose (SMD -0.07; 95% CI -0.20 to -0.06; **Figure S10**).Low heterogeneity among trials was discovered (I^2^ = 38%, P = 0.04).The funnel plot suggested the existence of asymmetry (Egger test, P = 0.095; **Figure S11**), indicating possible publication bias.

### Safety of Probiotics in IBS

**Figure 9:**
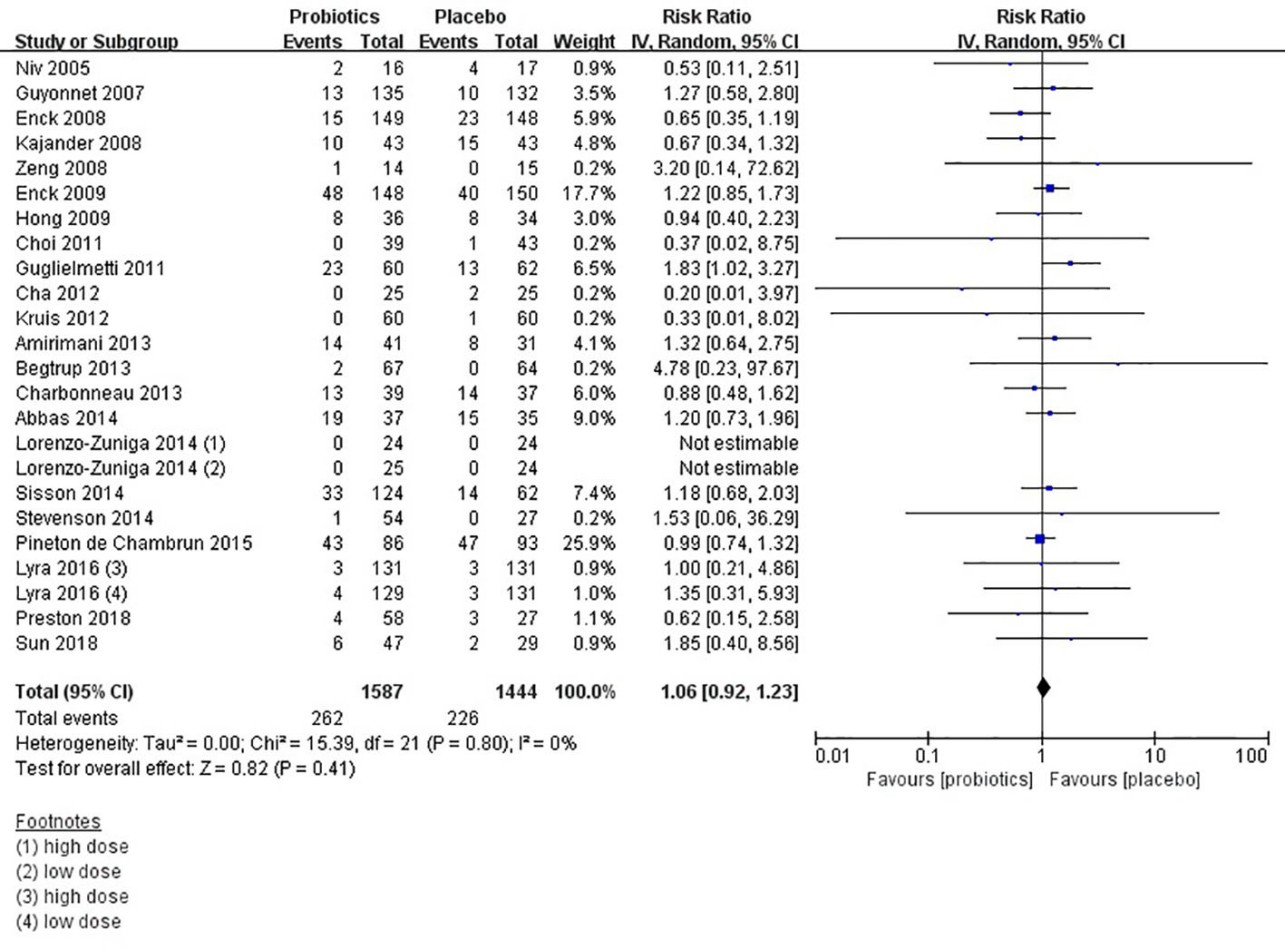
Forest plot of safety of probiotics in IBS.

Forty studies (Gade and Thorn, 1989; Niedzielin et al., 2001; Kim et al., 2003; Kajander et al., 2005; Kim et al., 2005; Niv et al., 2005; O'Mahony et al., 2005; Kim et al., 2006; Whorwell et al., 2006; Guyonnet et al., 2007; Enck et al., 2008; Kajander et al., 2008; Sinn et al., 2008; Zeng et al., 2008; Enck et al., 2009; Hong et al., 2009; Simrén et al., 2010; Choi et al., 2011; Guglielmetti et al., 2011; Michail and Kenche, 2011; Cha et al., 2012; Dapoigny et al., 2012; Ducrotte et al., 2012; Kruis et al., 2012; Amirimani et al., 2013; Begtrup et al., 2013; Charbonneau et al., 2013; Abbas et al., 2014; Lorenzo-Zuniga et al., 2014; Sisson et al., 2014; Stevenson et al., 2014; Yoon et al., 2014; Pineton de Chambrun et al., 2015; Lyra et al., 2016; Majeed et al., 2016; Spiller et al., 2016; Hod et al., 2017; Ishaque et al., 2018; Preston et al., 2018; Sun et al., 2018) provided safety-related data, which was assessed by adverse events. Fourteen trials (Gade and Thorn, 1989; Niedzielin et al., 2001; Kim et al., 2003; Kajander et al., 2005; Kim et al., 2005; Kim et al., 2006; Sinn et al., 2008; Simrén et al., 2010; Michail and Kenche, 2011; Dapoigny et al., 2012; Lorenzo-Zuniga et al., 2014; Yoon et al., 2015; Hod et al., 2017; Ishaque et al., 2018) reported that there were no adverse events. Four trials (O'Mahony et al., 2005; Whorwell et al., 2006; Majeed et al., 2016; Spiller et al., 2016) reported adverse events of both arms. Difference was detected between probiotics and placebo (RR = 1.07; 95% CI 0.92–1.24; **Figure 9**), with low heterogeneity (I^2^ = 0, P = 0.83). The funnel plot suggested no evidence of asymmetry (Egger test, P = 0.808; **Figure S12**). Probiotics seem to be safer than placebo in IBS patients.

## Discussion

Alterations of the intestinal microbiome could be relevant to IBS. Symptoms in IBS often developed after an infection, which was known as post-infectious IBS (Marshall et al., 2006; Marshall et al., 2007). Gut bacterial overgrowth may cause symptoms of IBS indistinguishable (Lin, 2004). Studies suggest that compared with the healthy group the colonic microbiome changes in IBS (Durban et al., 2013; Jalanka-Tuovinen et al., 2014). Despite there were many drugs and treatments for IBS, probiotics have shown beneficial (Simrén et al., 2013; Mozaffari et al., 2014). Probiotics may regulate immunity in IBS to protect the intestine (Major and Spiller, 2014). Probiotics also modify the gut microbiota, which improves some IBS symptoms, such as flatulence, bloating, and altered bowel habits (Jeffery et al., 2012; Tap et al., 2017).

### Summary of Main Results

Many pieces of evidence have suggested that probiotics may benefit IBS symptoms (Shavakhi et al., 2014; Stevenson et al., 2014; Yoon et al., 2014). However, the results of clinical trials have been conflicting. Our meta-analysis has indicated that probiotics may be beneficial and safe to improve symptoms of IBS compared with placebo. However, it was difficult to draw a precise conclusion as a result of the existence of significant heterogeneity and possible publication bias. We found that a shorter treatment duration (< 8 weeks) could reduce global IBS symptoms scores and bloating scores (Whorwell et al., 2006; Guyonnet et al., 2007; Drouault-Holowacz et al., 2008). As a chronic and recurrent disease (Sun et al., 2018), the improvement of IBS symptoms seems to be detected after a long time by taking probiotics continuously. However, according to current research shorter treatment duration seemed to be more beneficial. But due to many dropouts in the longer duration group, there may have an impact on research results, manifesting as greater improvement in the shorter duration group (Roberts et al., 2013). Although the use of single probiotics tended to have a beneficial effect on improving the bloating scores (Majeed et al., 2016; Spiller et al., 2016; Kim et al., 2018; Sun et al., 2018), it was unknown which strain or species was more beneficial than others. Using a high dose of probiotics may reduce abdominal pain scores and bloating scores (Yoon et al., 2014; Yoon et al., 2015; Kim et al., 2018). However, Lyra et al. tested two different doses (10^10^ CFU/D, and 10^9^ CFU/D) of Lactobacillus acidophilus NCFM and reported that none of the outcomes showed a dose-response effect (Lyra et al., 2016). Small differences of dosage may contribute to no effect of dose. Probiotics could benefit overall IBS symptoms improvement in North America, West Europe, Northern Europe, and East Asian.We also found that probiotics could reduce global IBS symptoms scores in North America, East Asian, and South Asian. More pieces of evidence are needed. Probiotics seemed safe for patients with irritable bowel syndrome (O'Mahony et al., 2005; Whorwell et al., 2006; Majeed et al., 2016; Spiller et al., 2016), but more long-term trials are required to prove it.

### Strengths and Weaknesses

Our meta-analysis is the first to assemble the efficacy and safety of probiotics for IBS patients with all diagnostic criteria by subgroup analyses of probiotic type, dose, treatment duration, and geographic position. We conducted this meta-analysis and systematic review using a rigorous and reproducible methodology. Two reviewers assessed eligibility and extracted data independently. The random-effects model was used to minimize the possibility of overestimating treatment results. We also tried to contact researchers of possibly eligible trials to get data. These comprehensive approaches included more than 3,300 IBS patients receiving probiotics treatment. Finally, subgroup analyses of probiotics type, dose, treatment duration, and geographic position were performed to evaluate the efficacy of treatment.

Our study has certain limitations. Bias risk of many studies was unknown, and the analysis shows considerable evidence of heterogeneity between trials. However, considering only studies with low bias risk, the positive effects remained. The number of studies on subgroup analyses of probiotics type, doses, and treatment duration was limited. It was not enough to detect significant differences in the efficacy of probiotics. In some studies, significant placebo effects have been found which can affect the results.

## Conclusions

In summary, this meta-analysis has demonstrated moderate evidence for the use and safety of probiotics in IBS. A shorter treatment duration (< 8 weeks) and a single probiotic may be more beneficial. Probiotics seem to be safe for patients with irritable bowel syndrome. There is still a need for more clinical trials. Finally, probiotics may be a beneficial therapy for IBS patients.

## Data Availability Statement

All datasets generated for this study are included in the article/**Supplementary Material**.

## Author Contributions

LZ and BL conceived and designed this study. BL and LL searched and selected studies. HD and JG extracted essential information. BL and HS assessed the risk of bias. BL and HD performed statistical analyses. BL and HS interpreted the pooled results. BL, LL, and LZ drafted the manuscript. All authors approved the final manuscript.

## Funding

This research was supported by the Youth Foundation of 960th Hospital of the PLA with a unique identifier of 2017QN03.

## Conflict of Interest

The authors declare that the research was conducted in the absence of any commercial or financial relationships that could be construed as a potential conflict of interest.
