# Supplementary material for: Efficacy and Safety of Probiotics in Irritable Bowel Syndrome: A Systematic Review and Meta-Analysis
Source: Front Pharmacol. 2020 Apr 3;11:332. doi: 10.3389/fphar.2020.00332 (PMC7147251; doi:10.3389/fphar.2020.00332)
Supplement: Supplementary file 1 [file DataSheet_1.docx]

**SEARCHING STRATEGY**

**Term**

("probiotics"[MeSH Terms] OR "probiotics"[Title/Abstract]) AND ("irritable bowel syndrome"[MeSH Terms] OR "irritable bowel syndrome"[Title/Abstract]) AND ("randomized controlled trial" [pt] OR "randomized controlled trial" [tiab])

**PubMed**

((((((((((((((("Irritable Bowel Syndrome"[Mesh]) OR Irritable Bowel Syndromes[Title/Abstract])) OR “Syndrome, Irritable Bowel”[Title/Abstract]) OR “Syndromes, Irritable Bowel”[Title/Abstract])) OR “Colon, Irritable”[Title/Abstract])) OR Irritable Colon[Title/Abstract]) OR “Colitis, Mucous”[Title/Abstract])) OR “Colitides, Mucous”[Title/Abstract])) OR Mucous Colitides[Title/Abstract]) OR Mucous Colitis[Title/Abstract]) Sort by: PublicationDate 5783

"Colonic Diseases"[Mesh] OR "Colonic Diseases, Functional"[Mesh] Sort by: PublicationDate 210769

((((((((spastic colon[Title/Abstract]) OR irritable colon[Title/Abstract]) OR irritable bowel[Title/Abstract]) OR functional bowel[Title/Abstract]) OR colonic disease[Title/Abstract]) OR colonic diseases[Title/Abstract]) OR IBS[Title/Abstract]) OR gastrointestinal syndrome[Title/Abstract]) OR gastrointestinal syndromes[Title/Abstract] Sort by: PublicationDate 14511

(((((((((((spastic colon[Title/Abstract]) OR irritable colon[Title/Abstract]) OR irritable bowel[Title/Abstract]) OR functional bowel[Title/Abstract]) OR colonic disease[Title/Abstract]) OR colonic diseases[Title/Abstract]) OR IBS[Title/Abstract]) OR gastrointestinal syndrome[Title/Abstract]) OR gastrointestinal syndromes[Title/Abstract])) OR ("Colonic Diseases"[Mesh] OR "Colonic Diseases, Functional"[Mesh])) OR (((((((((((((((("Irritable Bowel Syndrome"[Mesh]) OR Irritable Bowel Syndromes[Title/Abstract])) OR “Syndrome, Irritable Bowel”[Title/Abstract]) OR “Syndromes, Irritable Bowel”[Title/Abstract])) OR “Colon, Irritable”[Title/Abstract])) OR Irritable Colon[Title/Abstract]) OR “Colitis, Mucous”[Title/Abstract])) OR “Colitides, Mucous”[Title/Abstract])) OR Mucous Colitides[Title/Abstract]) OR Mucous Colitis[Title/Abstract])) Sort by: PublicationDate 216476

"Probiotics"[Mesh] 11450

"Synbiotics"[Mesh] 237

"Lactobacillus"[Mesh] 23672

"Lactococcus"[Mesh] 4431

"Lactococcus lactis"[Mesh] 3826

"Bifidobacterium"[Mesh] 4600

"Yeast, Dried"[Mesh] OR "Saccharomyces cerevisiae"[Mesh] OR "Yeasts"[Mesh] 175087

"Yogurt"[Mesh] 1599

"Saccharomyces"[Mesh] 99134

"Streptococcus"[Mesh] OR "Streptococcus thermophilus"[Mesh] 70469

"Leuconostoc"[Mesh] 1709

"Pediococcus"[Mesh] 883

"Enterococcus"[Mesh] 16722

"Bacillus"[Mesh] OR "Bacillus subtilis"[Mesh] 59922

"Clostridium"[Mesh] 27184

(((((((((((((((((((((((Probiotics[Title/Abstract]) OR Probiotic[Title/Abstract]) OR probiotic agent[Title/Abstract]) OR synbiotic agent[Title/Abstract]) OR symbiotic[Title/Abstract]) OR symbiotics[Title/Abstract]) OR Lactobacillus[Title/Abstract]) OR lactobacil[Title/Abstract]) OR lactococcus[Title/Abstract]) OR bifidobacterium[Title/Abstract]) OR bifidus[Title/Abstract]) OR bifidobacter[Title/Abstract]) OR yeast[Title/Abstract]) OR yogurt[Title/Abstract]) OR saccharomyces[Title/Abstract]) OR streptococcus[Title/Abstract]) OR leuconostoc[Title/Abstract]) OR Leukonostoc[Title/Abstract]) OR Pediococcus[Title/Abstract]) OR Enterococcus[Title/Abstract]) OR Bacillus subtilis[Title/Abstract]) OR Bacillus clausii[Title/Abstract]) OR Bacillus[Title/Abstract]) OR clostridium[Title/Abstract] 396380

(((((((((((((((((((((((((((((((((((((((Probiotics[Title/Abstract]) OR Probiotic[Title/Abstract]) OR probiotic agent[Title/Abstract]) OR synbiotic agent[Title/Abstract]) OR symbiotic[Title/Abstract]) OR symbiotics[Title/Abstract]) OR Lactobacillus[Title/Abstract]) OR lactobacil[Title/Abstract]) OR lactococcus[Title/Abstract]) OR bifidobacterium[Title/Abstract]) OR bifidus[Title/Abstract]) OR bifidobacter[Title/Abstract]) OR yeast[Title/Abstract]) OR yogurt[Title/Abstract]) OR saccharomyces[Title/Abstract]) OR streptococcus[Title/Abstract]) OR leuconostoc[Title/Abstract]) OR Leukonostoc[Title/Abstract]) OR Pediococcus[Title/Abstract]) OR Enterococcus[Title/Abstract]) OR Bacillus subtilis[Title/Abstract]) OR Bacillus clausii[Title/Abstract]) OR Bacillus[Title/Abstract]) OR clostridium[Title/Abstract])) OR "Clostridium"[Mesh]) OR ("Bacillus"[Mesh] OR "Bacillus subtilis"[Mesh])) OR "Enterococcus"[Mesh]) OR "Pediococcus"[Mesh]) OR "Leuconostoc"[Mesh]) OR ("Streptococcus"[Mesh] OR "Streptococcus thermophilus"[Mesh])) OR "Saccharomyces"[Mesh]) OR "Yogurt"[Mesh]) OR ("Yeast, Dried"[Mesh] OR "Saccharomyces cerevisiae"[Mesh] OR "Yeasts"[Mesh])) OR "Bifidobacterium"[Mesh]) OR "Lactococcus lactis"[Mesh]) OR "Lactococcus"[Mesh]) OR "Lactobacillus"[Mesh]) OR "Synbiotics"[Mesh]) OR "Probiotics"[Mesh] 515064

(((((((randomized controlled trial [pt]) OR controlled clinical trial [pt]) OR randomized [tiab]) OR placebo [tiab]) OR drug therapy [sh]) OR randomly [tiab]) OR trial [tiab]) OR groups [tiab] Sort by: PublicationDate 3920789

(animals [mh] NOT humans [mh]) Sort by: PublicationDate 4271572

(((((((((randomized controlled trial [pt]) OR controlled clinical trial [pt]) OR randomized [tiab]) OR placebo [tiab]) OR drug therapy [sh]) OR randomly [tiab]) OR trial [tiab]) OR groups [tiab])) NOT ((animals [mh] NOT humans [mh])) Sort by: PublicationDate 3386301

(((((((((((((((spastic colon[Title/Abstract]) OR irritable colon[Title/Abstract]) OR irritable bowel[Title/Abstract]) OR functional bowel[Title/Abstract]) OR colonic disease[Title/Abstract]) OR colonic diseases[Title/Abstract]) OR IBS[Title/Abstract]) OR gastrointestinal syndrome[Title/Abstract]) OR gastrointestinal syndromes[Title/Abstract])) OR ("Colonic Diseases"[Mesh] OR "Colonic Diseases, Functional"[Mesh])) OR (((((((((((((((("Irritable Bowel Syndrome"[Mesh]) OR Irritable Bowel Syndromes[Title/Abstract])) OR “Syndrome, Irritable Bowel”[Title/Abstract]) OR “Syndromes, Irritable Bowel”[Title/Abstract])) OR “Colon, Irritable”[Title/Abstract])) OR Irritable Colon[Title/Abstract]) OR “Colitis, Mucous”[Title/Abstract])) OR “Colitides, Mucous”[Title/Abstract])) OR Mucous Colitides[Title/Abstract]) OR Mucous Colitis[Title/Abstract]))))) AND ((((((((((((randomized controlled trial [pt]) OR controlled clinical trial [pt]) OR randomized [tiab]) OR placebo [tiab]) OR drug therapy [sh]) OR randomly [tiab]) OR trial [tiab]) OR groups [tiab])) NOT ((animals [mh] NOT humans [mh])))))) AND (((((((((((((((((((((((((((((((((((((((((Probiotics[Title/Abstract]) OR Probiotic[Title/Abstract]) OR probiotic agent[Title/Abstract]) OR synbiotic agent[Title/Abstract]) OR symbiotic[Title/Abstract]) OR symbiotics[Title/Abstract]) OR Lactobacillus[Title/Abstract]) OR lactobacil[Title/Abstract]) OR lactococcus[Title/Abstract]) OR bifidobacterium[Title/Abstract]) OR bifidus[Title/Abstract]) OR bifidobacter[Title/Abstract]) OR yeast[Title/Abstract]) OR yogurt[Title/Abstract]) OR saccharomyces[Title/Abstract]) OR streptococcus[Title/Abstract]) OR leuconostoc[Title/Abstract]) OR Leukonostoc[Title/Abstract]) OR Pediococcus[Title/Abstract]) OR Enterococcus[Title/Abstract]) OR Bacillus subtilis[Title/Abstract]) OR Bacillus clausii[Title/Abstract]) OR Bacillus[Title/Abstract]) OR clostridium[Title/Abstract])) OR "Clostridium"[Mesh]) OR ("Bacillus"[Mesh] OR "Bacillus subtilis"[Mesh])) OR "Enterococcus"[Mesh]) OR "Pediococcus"[Mesh]) OR "Leuconostoc"[Mesh]) OR ("Streptococcus"[Mesh] OR "Streptococcus thermophilus"[Mesh])) OR "Saccharomyces"[Mesh]) OR "Yogurt"[Mesh]) OR ("Yeast, Dried"[Mesh] OR "Saccharomyces cerevisiae"[Mesh] OR "Yeasts"[Mesh])) OR "Bifidobacterium"[Mesh]) OR "Lactococcus lactis"[Mesh]) OR "Lactococcus"[Mesh]) OR "Lactobacillus"[Mesh]) OR "Synbiotics"[Mesh]) OR "Probiotics"[Mesh])) Sort by: PublicationDate 2233**Embase**

'irritable colon'/exp 20212

'gastrointestinal disease'/exp 84290

'colonic diseases':ab,ti 446

'colonic diseases, functional':ab,ti 3

'irritable bowel syndrome':ab,ti 15389

'spastic colon':ab,ti 92

'irritable colon':ab,ti 572

'irritable bowel':ab,ti 15897

'functional bowel':ab,ti 1304

'colonic disease':ab,ti 1138

'colonic diseases':ab,ti 446

'ibs':ab,ti 11656

'gastrointestinal syndrome':ab,ti 177

'gastrointestinal syndromes':ab,ti 104

'irritable bowel syndromes':ab,ti 29

'syndrome, irritable bowel':ab,ti 57

'syndromes, irritable bowel':ab,ti 4

'colon, irritable':ab,ti 86

'colitis mucous':ab,ti 3

'colitides, mucous':ab,ti 0

'mucous colitides':ab,ti 0

'mucous colitis':ab,ti 14

'irritable colon'/exp OR 'gastrointestinal disease'/exp OR 'colonic diseases, functional':ab,ti OR 'irritable bowel syndrome':ab,ti OR 'spastic colon':ab,ti OR 'irritable colon':ab,ti OR 'irritable bowel':ab,ti OR 'functional bowel':ab,ti OR 'colonic disease':ab,ti OR 'colonic diseases':ab,ti OR 'ibs':ab,ti OR 'gastrointestinal syndrome':ab,ti OR 'gastrointestinal syndromes':ab,ti OR 'irritable bowel syndromes':ab,ti OR 'syndrome, irritable bowel':ab,ti OR 'syndromes, irritable bowel':ab,ti OR 'colon, irritable':ab,ti OR 'colitis mucous':ab,ti OR 'colitides, mucous':ab,ti OR 'mucous colitides':ab,ti OR 'mucous colitis':ab,ti 107513

'probiotic agent'/exp 23118

'synbiotic agent'/exp 878

'lactobacillus'/exp 35538

'lactococcus'/exp 6443

'lactococcus'/exp 6443

'yeast'/exp 69542

'yoghurt'/exp 3933

'saccharomyces'/exp 93213

'streptococcus'/exp 116280

'leuconostoc'/exp 2459

'leuconostoc lactis'/exp 61

'pediococcus'/exp 1411

'enterococcus'/exp 38908

'bacillus subtilis'/exp 38160

'bacillus'/exp 80239

'clostridium'/exp 42091

'probiotic agent'/exp OR 'synbiotic agent'/exp OR 'lactobacillus'/exp OR 'lactococcus'/exp OR 'yeast'/exp OR 'yoghurt'/exp OR 'saccharomyces'/exp OR 'streptococcus'/exp OR 'leuconostoc'/exp OR 'leuconostoc lactis'/exp OR 'pediococcus'/exp OR 'enterococcus'/exp OR 'bacillus subtilis'/exp OR 'bacillus'/exp OR 'clostridium'/exp 447117

probiotics:ab,ti 11682

probiotic:ab,ti 12643

'probiotic agent':ab,ti 82

'synbiotic agent':ab,ti 0

'symbiotic':ab,ti 10501

'symbiotics':ab,ti 62

'lactobacillus':ab,ti 24780

'lactobacil':ab,ti 6

'lactococcus':ab,ti 5622

'bifidobacterium':ab,ti 6411

'bifidus':ab,ti 390

'yeast':ab,ti 167473

'yoghurt':ab,ti 1341

'saccharomyces':ab,ti 68541

'streptococcus':ab,ti 71252

'leuconostoc':ab,ti 2341

'leukonostoc':ab,ti 1

'pediococcus':ab,ti 1377

'enterococcus':ab,ti 20249

'bacillus subtilis':ab,ti 28358

'bacillus clausii':ab,ti 111

'bacillus':ab,ti 80636

'clostridium':ab,ti 37018

'probiotic agent'/exp OR 'synbiotic agent'/exp OR 'lactobacillus'/exp OR 'lactococcus'/exp OR 'yeast'/exp OR 'yoghurt'/exp OR 'saccharomyces'/exp OR 'streptococcus'/exp OR 'leuconostoc'/exp OR 'leuconostoc lactis'/exp OR 'pediococcus'/exp OR 'enterococcus'/exp OR 'bacillus subtilis'/exp OR 'bacillus'/exp OR 'clostridium'/exp OR probiotics:ab,ti OR probiotic:ab,ti OR 'probiotic agent':ab,ti OR 'synbiotic agent':ab,ti OR 'symbiotic':ab,ti OR 'symbiotics':ab,ti OR 'lactobacillus':ab,ti OR 'lactobacil':ab,ti OR 'lactococcus':ab,ti OR 'bifidobacterium':ab,ti OR 'bifidus':ab,ti OR 'yeast':ab,ti OR 'yoghurt':ab,ti OR 'saccharomyces':ab,ti OR 'streptococcus':ab,ti OR 'leuconostoc':ab,ti OR 'leukonostoc':ab,ti OR 'pediococcus':ab,ti OR 'enterococcus':ab,ti OR 'bacillus subtilis':ab,ti OR 'bacillus clausii':ab,ti OR 'bacillus':ab,ti OR 'clostridium':ab,ti 577776

'clinical trial'/exp OR 'clinical trial' OR 'randomized controlled trial'/exp OR 'randomized controlled trial' OR 'randomization'/exp OR 'randomization' OR 'single blind procedure'/exp OR 'single blind procedure' OR 'double blind procedure'/exp OR 'double blind procedure' OR 'crossover procedure'/exp OR 'crossover procedure' OR 'placebo'/exp OR 'placebo' OR 'prospective study'/exp OR 'prospective study' OR 'randomi?ed controlled' NEXT/1 trial* OR rct OR 'randomly allocated' OR 'allocated randomly' OR 'random allocation'/exp OR 'random allocation' OR allocated NEAR/2 random OR single NEXT/1 blind* OR double NEXT/1 blind* OR (treble OR triple) NEAR/1 blind* OR placebo* 1998205

'irritable colon'/exp OR 'gastrointestinal disease'/exp OR 'colonic diseases, functional':ab,ti OR 'irritable bowel syndrome':ab,ti OR 'spastic colon':ab,ti OR 'irritable colon':ab,ti OR 'irritable bowel':ab,ti OR 'functional bowel':ab,ti OR 'colonic disease':ab,ti OR 'colonic diseases':ab,ti OR 'ibs':ab,ti OR 'gastrointestinal syndrome':ab,ti OR 'gastrointestinal syndromes':ab,ti OR 'irritable bowel syndromes':ab,ti OR 'syndrome, irritable bowel':ab,ti OR 'syndromes, irritable bowel':ab,ti OR 'colon, irritable':ab,ti OR 'colitis mucous':ab,ti OR 'colitides, mucous':ab,ti OR 'mucous colitides':ab,ti OR 'mucous colitis':ab,ti AND ('probiotic agent'/exp OR 'synbiotic agent'/exp OR 'lactobacillus'/exp OR 'lactococcus'/exp OR 'yeast'/exp OR 'yoghurt'/exp OR 'saccharomyces'/exp OR 'streptococcus'/exp OR 'leuconostoc'/exp OR 'leuconostoc lactis'/exp OR 'pediococcus'/exp OR 'enterococcus'/exp OR 'bacillus subtilis'/exp OR 'bacillus'/exp OR 'clostridium'/exp OR probiotics:ab,ti OR probiotic:ab,ti OR 'probiotic agent':ab,ti OR 'synbiotic agent':ab,ti OR 'symbiotic':ab,ti OR 'symbiotics':ab,ti OR 'lactobacillus':ab,ti OR 'lactobacil':ab,ti OR 'lactococcus':ab,ti OR 'bifidobacterium':ab,ti OR 'bifidus':ab,ti OR 'yeast':ab,ti OR 'yoghurt':ab,ti OR 'saccharomyces':ab,ti OR 'streptococcus':ab,ti OR 'leuconostoc':ab,ti OR 'leukonostoc':ab,ti OR 'pediococcus':ab,ti OR 'enterococcus':ab,ti OR 'bacillus subtilis':ab,ti OR 'bacillus clausii':ab,ti OR 'bacillus':ab,ti OR 'clostridium':ab,ti) AND ('clinical trial'/exp OR 'clinical trial' OR 'randomized controlled trial'/exp OR 'randomized controlled trial' OR 'randomization'/exp OR 'randomization' OR 'single blind procedure'/exp OR 'single blind procedure' OR 'double blind procedure'/exp OR 'double blind procedure' OR 'crossover procedure'/exp OR 'crossover procedure' OR 'placebo'/exp OR 'placebo' OR 'prospective study'/exp OR 'prospective study' OR 'randomi?ed controlled' NEXT/1 trial* OR rct OR 'randomly allocated' OR 'allocated randomly' OR 'random allocation'/exp OR 'random allocation' OR allocated NEAR/2 random OR single NEXT/1 blind* OR double NEXT/1 blind* OR (treble OR triple) NEAR/1 blind* OR placebo*) 1522

**Cochrane**

#1 MeSH descriptor: [Colonic Diseases] explode all trees 7692

#2 MeSH descriptor: [Colonic Diseases, Functional] explode all trees 936

#3 MeSH descriptor: [Irritable Bowel Syndrome] explode all trees 604

#4 spastic colon:ti,ab,kw or irritable colon:ti,ab,kw or irritable bowel:ti,ab,kw or functional bowel:ti,ab,kw or colonic disease:ti,ab,kw (Word variations have been searched) 3773

#5 colonic diseases:ti,ab,kw or IBS:ti,ab,kw or gastrointestinal syndrome:ti,ab,kw or gastrointestinal syndromes:ti,ab,kw (Word variations have been searched) 4349

#6 Irritable Bowel Syndromes:ti,ab,kw or "Syndrome, Irritable Bowel":ti,ab,kw or "Syndromes, Irritable Bowel":ti,ab,kw or "Colon, Irritable":ti,ab,kw or "Colitis, Mucous":ti,ab,kw (Word variations have been searched) 1809

#7 "Colitides, Mucous":ti,ab,kw or Mucous Colitides:ti,ab,kw or Mucous Colitis:ti,ab,kw (Word variations have been searched) 15

#8 #1 or #2 or #3 or #4 or #5 or #6 or #7 11142

#9 MeSH descriptor: [Clostridium] explode all trees 314

#10 MeSH descriptor: [Bacillus] explode all trees 74

#11 MeSH descriptor: [Bacillus subtilis] explode all trees 23

#12 MeSH descriptor: [Enterococcus] explode all trees 255

#13 MeSH descriptor: [Pediococcus] explode all trees 5

#14 MeSH descriptor: [Leuconostoc] explode all trees 2

#15 MeSH descriptor: [Streptococcus] explode all trees 1664

#16 MeSH descriptor: [Streptococcus thermophilus] explode all trees 44

#17 MeSH descriptor: [Saccharomyces] explode all trees 148

#18 MeSH descriptor: [Yogurt] explode all trees 284

#19 MeSH descriptor: [Yeast, Dried] explode all trees 42

#20 MeSH descriptor: [Saccharomyces cerevisiae] explode all trees 70

#21 MeSH descriptor: [Yeasts] explode all trees 688

#22 MeSH descriptor: [Bifidobacterium] explode all trees 512

#23 MeSH descriptor: [Lactococcus lactis] explode all trees 10

#24 MeSH descriptor: [Lactococcus] explode all trees 11

#25 MeSH descriptor: [Lactobacillus] explode all trees 1248

#26 MeSH descriptor: [Synbiotics] explode all trees 72

#27 MeSH descriptor: [Probiotics] explode all trees 1530

#28 Probiotics:ti,ab,kw or Probiotic:ti,ab,kw or probiotic agent:ti,ab,kw or synbiotic agent:ti,ab,kw or synbiotic:ti,ab,kw (Word variations have been searched) 2898

#29 symbiotics:ti,ab,kw or lactobacillus:ti,ab,kw or lactobacil:ti,ab,kw or lactococcus:ti,ab,kw or bifidobacterium:ti,ab,kw (Word variations have been searched) 2780

#30 bifidus:ti,ab,kw or bifidus:ti,ab,kw or yeast:ti,ab,kw or yogurt:ti,ab,kw or saccharomyces:ti,ab,kw (Word variations have been searched) 1729

#31 streptococcus:ti,ab,kw or leuconostoc:ti,ab,kw or Leukonostoc:ti,ab,kw or pediococcus:ti,ab,kw or Enterococcus:ti,ab,kw (Word variations have been searched) 4287

#32 Bacillus subtilis:ti,ab,kw or Bacillus clausii:ti,ab,kw or Bacillus:ti,ab,kw or clostridium:ti,ab,kw (Word variations have been searched) 2108

#33 #9 or #10 or #11 or #12 or #13 or #14 or #15 or #16 or #17 or #18 or #19 or #20 or #21 or #22 or #23 or #24 or #25 or #26 or #27 or #28 or #29 or #30 or #31 or #32 10773

#34 #8 and #33 451
