# Supplementary figures and images for: Efficacy and Safety of Probiotics in Irritable Bowel Syndrome: A Systematic Review and Meta-Analysis
Source: Front Pharmacol. 2020 Apr 3;11:332. doi: 10.3389/fphar.2020.00332 (PMC7147251; doi:10.3389/fphar.2020.00332)

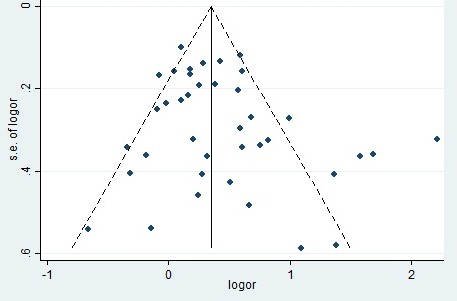

Supplement: Supplementary file 2 [file Image_1.jpeg]

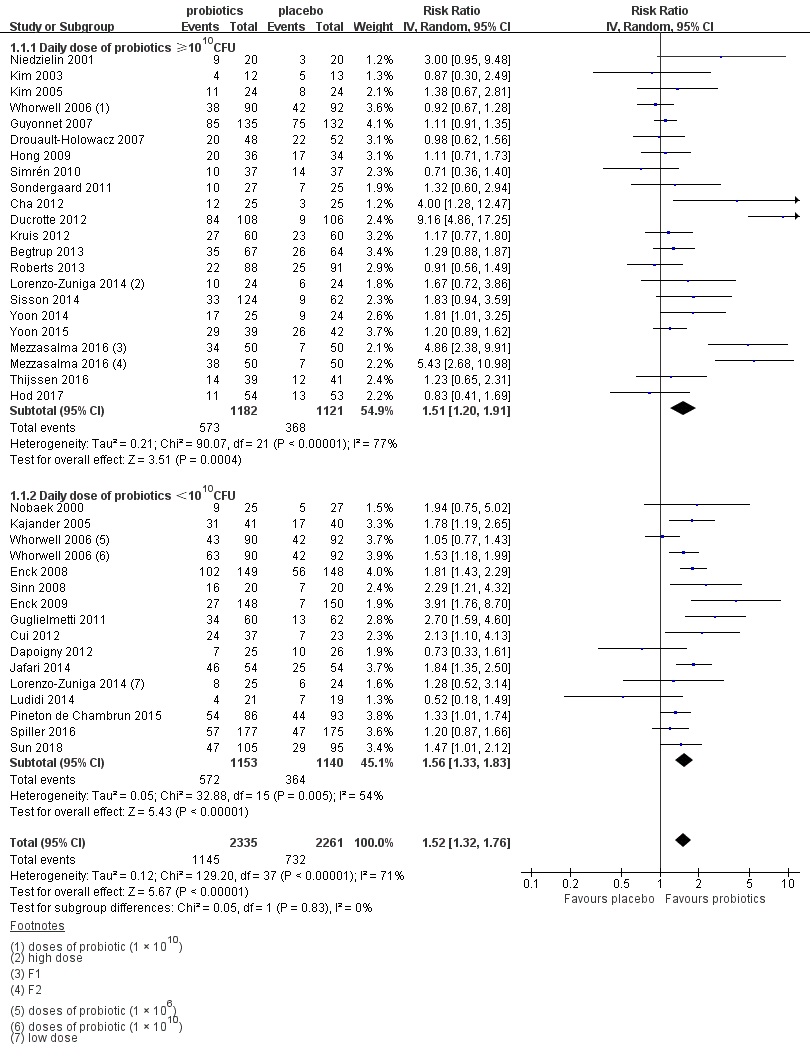

Supplement: Supplementary file 3 [file Image_2.jpeg]

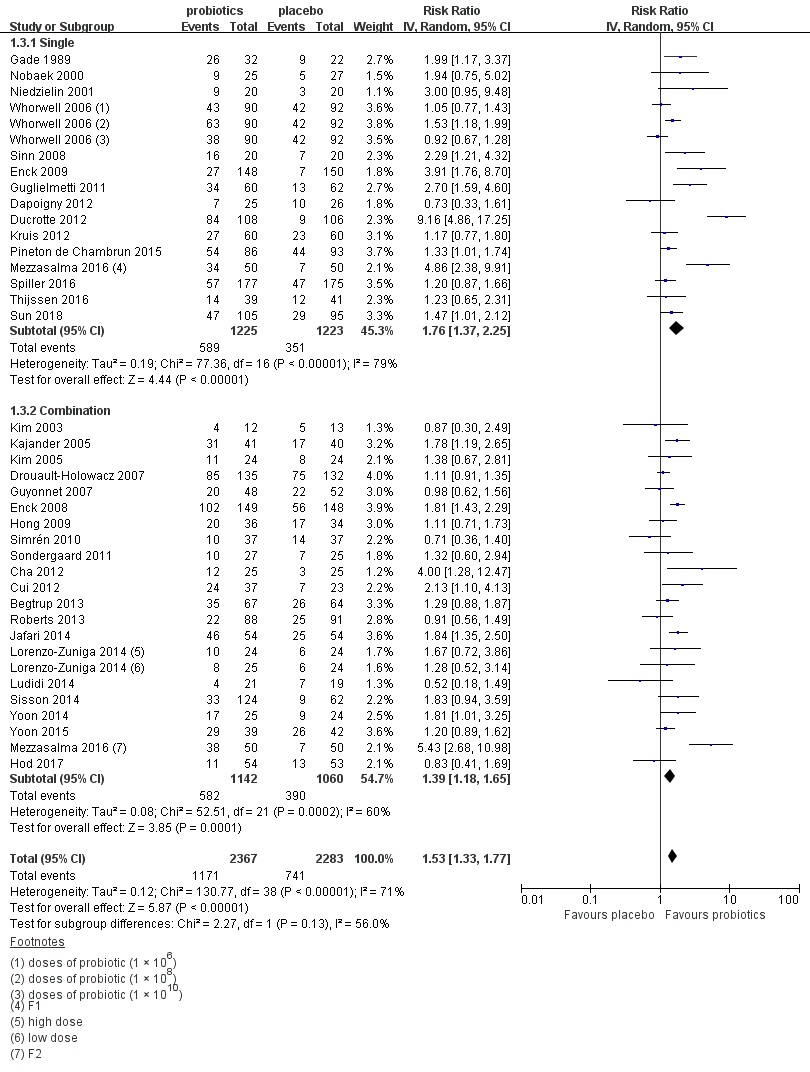

Supplement: Supplementary file 4 [file Image_3.jpeg]

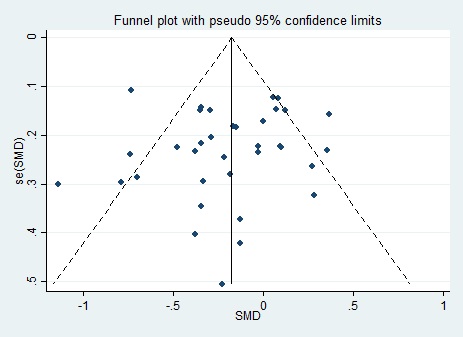

Supplement: Supplementary file 5 [file Image_4.jpeg]

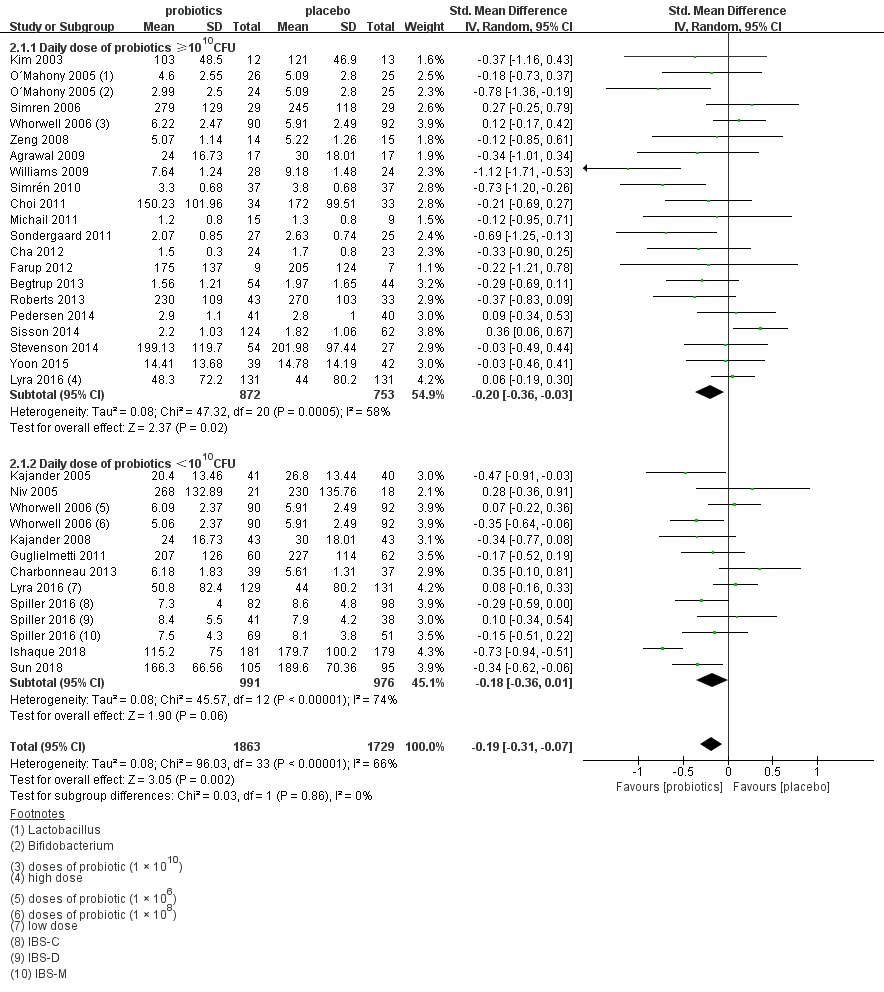

Supplement: Supplementary file 6 [file Image_5.jpeg]

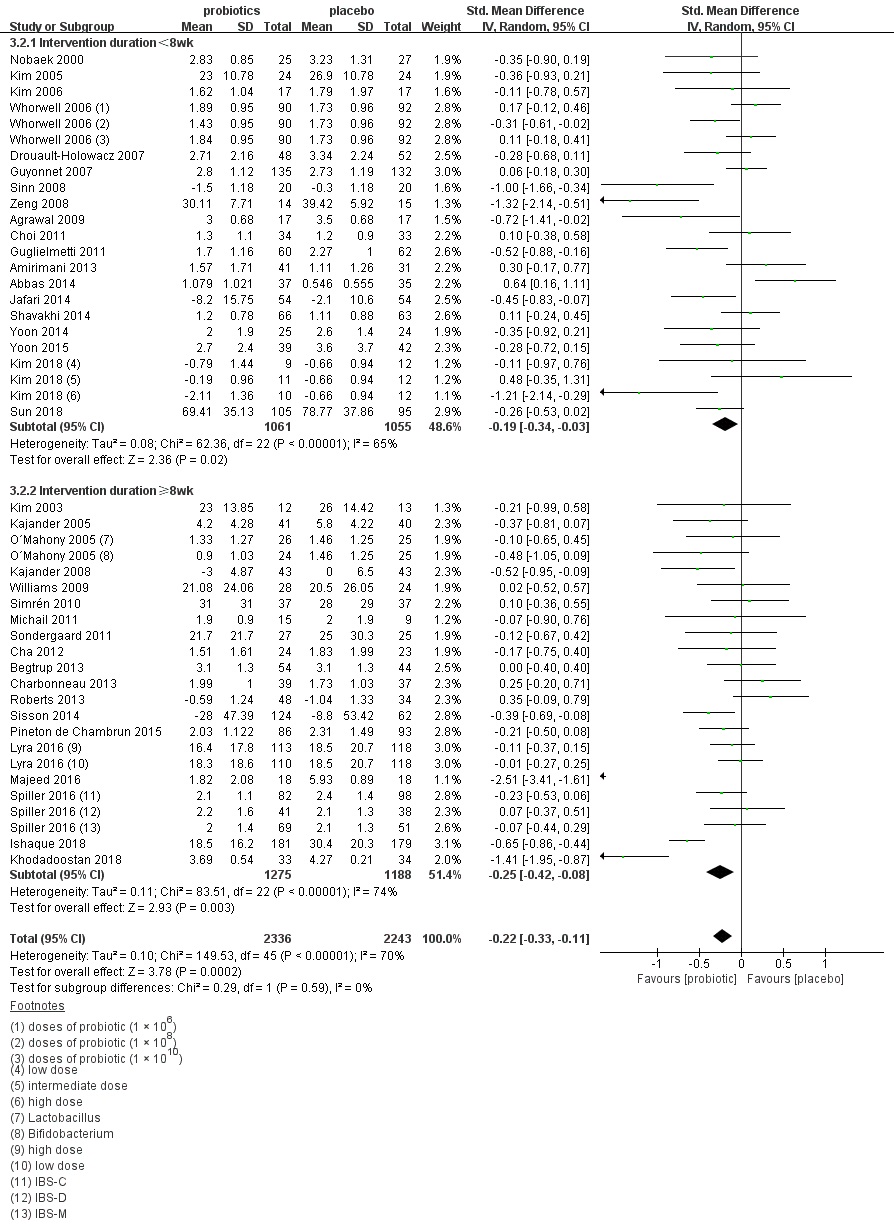

Supplement: Supplementary file 7 [file Image_6.jpeg]

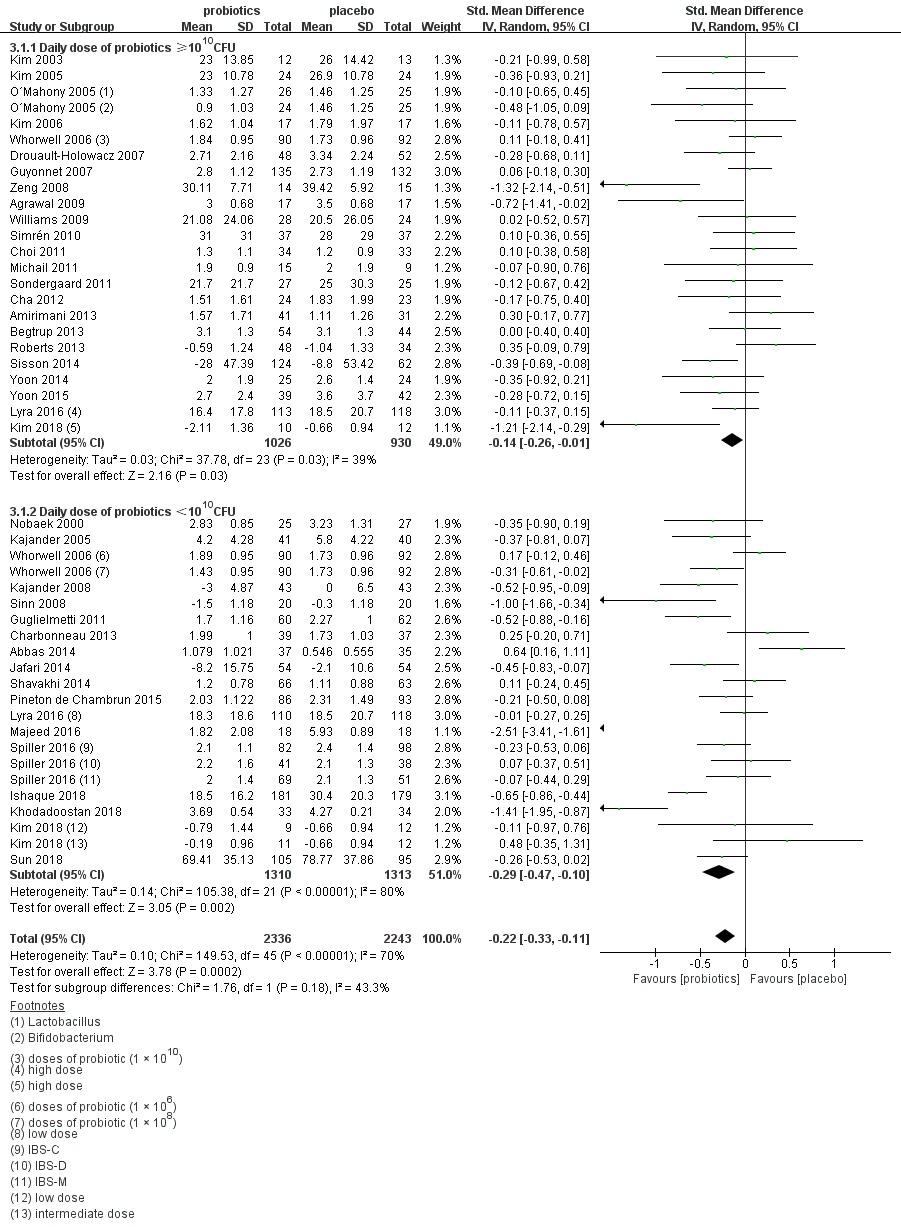

Supplement: Supplementary file 8 [file Image_7.jpeg]

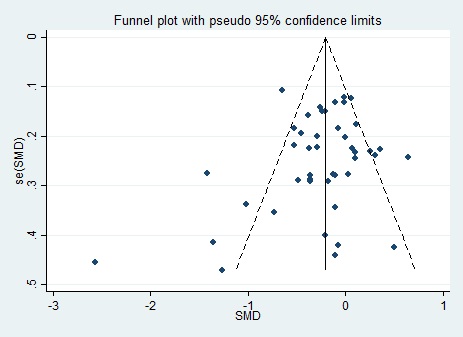

Supplement: Supplementary file 9 [file Image_8.jpeg]

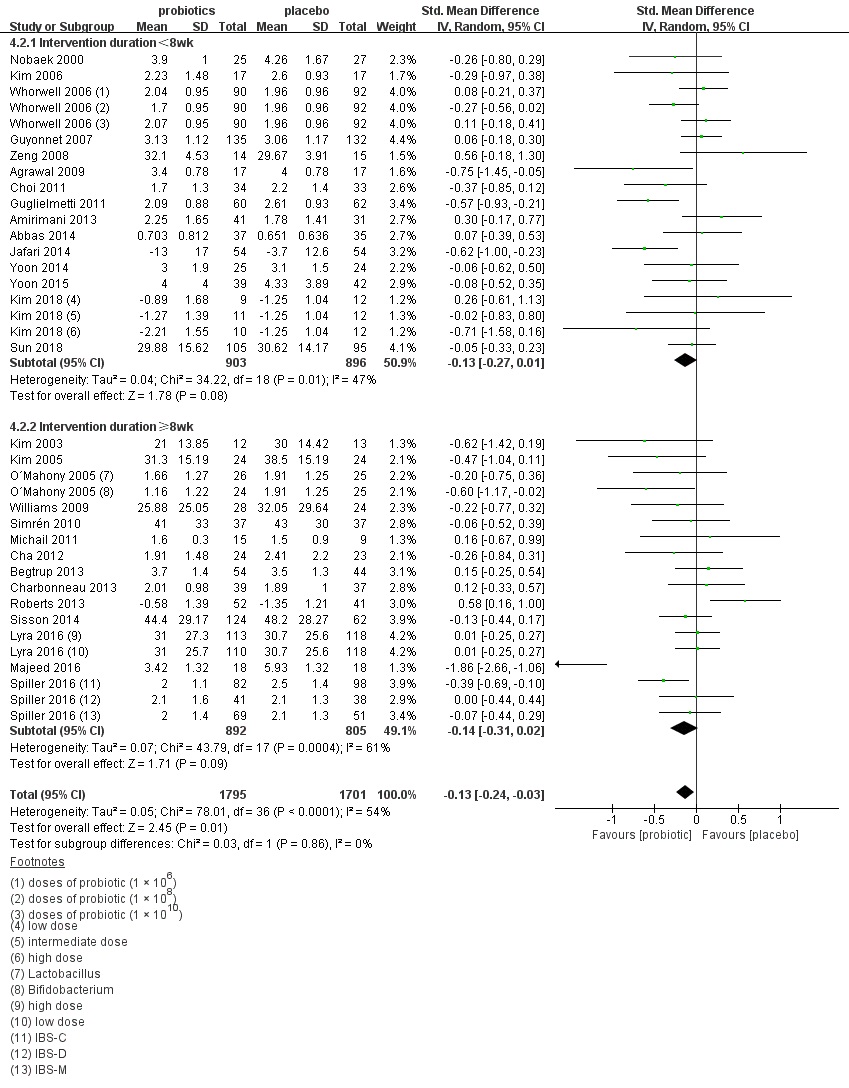

Supplement: Supplementary file 10 [file Image_9.jpeg]

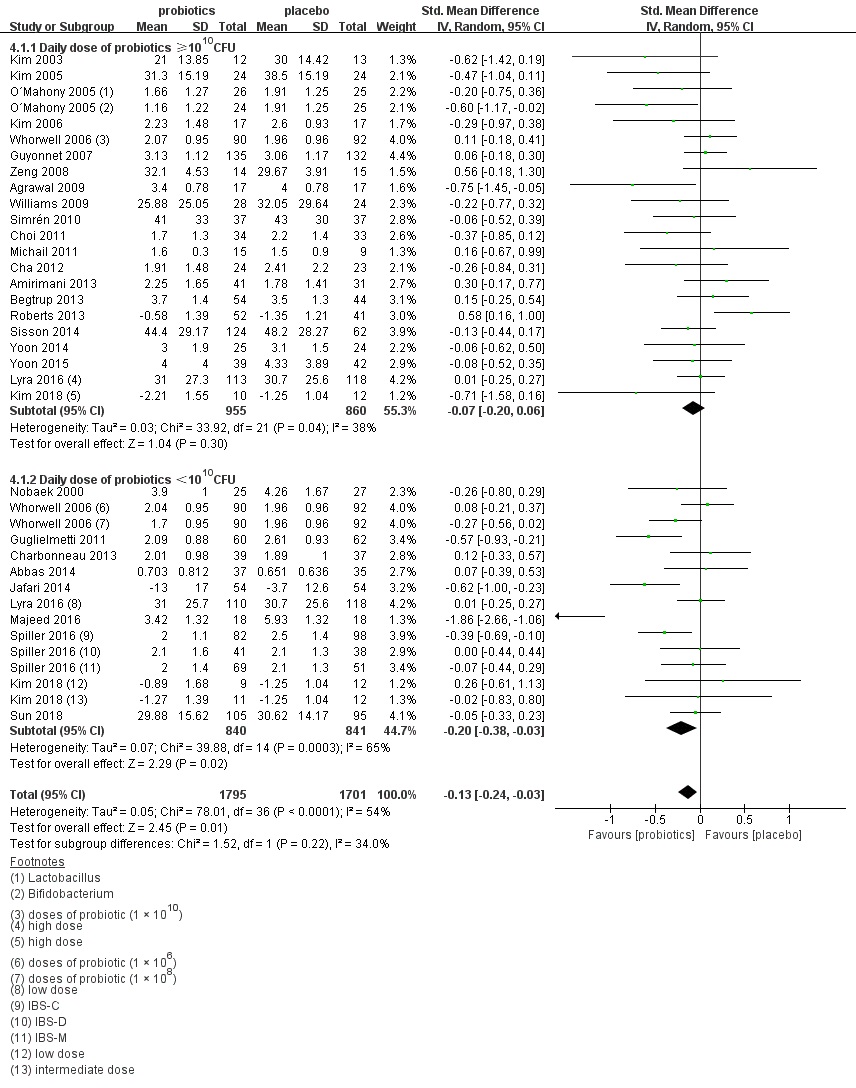

Supplement: Supplementary file 11 [file Image_10.jpeg]

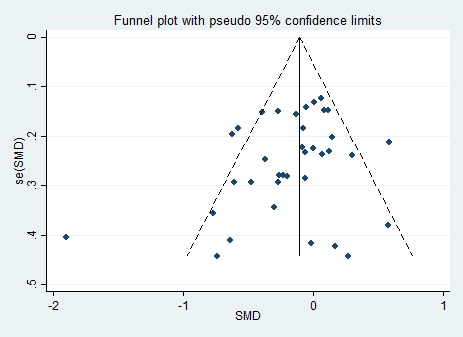

Supplement: Supplementary file 12 [file Image_11.jpeg]

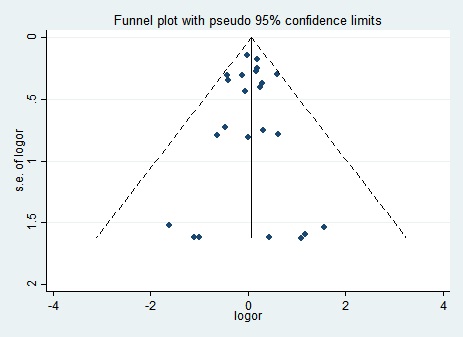

Supplement: Supplementary file 13 [file Image_12.jpeg]
